# Supplementary material for: Poor school attendance and exclusion: a systematic review protocol on educational risk factors for self-harm and suicidal behaviours
Source: BMJ Open. 2018 Dec 14;8(12):e023953. doi: 10.1136/bmjopen-2018-023953 (PMC6303662; doi:10.1136/bmjopen-2018-023953)
Supplement: Supplementary file 2 [file bmjopen-2018-023953supp002.pdf]

Search Strategies

PsycINFO

1. Child*.tw
2. Adolescen*.tw
3. Student*.tw
4. Exp Students/
5. Youth*.tw
6. Pupil*.tw
7. Schoolchild*.tw
8. (young adj (people or person).tw
9. teen*.tw
10. p?diatric.tw
11. exp Elementary School Students /
12. exp Suicide/
13. exp Suicide, Attempted/
14. exp Suicidal Ideation/
15. exp Drug Overdose/
16. exp Self-injurious Behaviour or exp Self Mutilation/
17. (autoaggress* or auto aggress* or automutilat* or cutt* or overdose* or (self adj2 cut*) or selfdestruct* or self destruct* or selfharm* or self harm* or selfimmolat* or self immolat* or selfinflict* or self inflict* or selfinjur* or self injur* or selfmutilat* or self mutilat* or selfpoison* or self poison* or suicide*).tw
18. exp school suspension/
19. exp school expulsion/
20. exp Educational Attainment Level/
21. (manage* adj move*).tw
22. (terminat* adj5 school*).tw
23. (exclusion or exclude*).tw
24. ‘failure to complete’.tw
25. expulsion.tw
26. suspension.tw
27. expel*.tw
28. ((school* or kindergarten or nursery or education) adj4 (attend* or non-attend* or refus* or absen* or school phobi* or truan*).tw
29. 1 or 2 or 3 or 4 or 5 or 6 or 7 or 8 or 9 or 10 or 11
30. 12 or 13 or 14 or 15 or 16 or 17
31. 18 or 19 or 20 or 21 or 22 or 23 or 24 or 25 or 26 or 27 or 28
32. 29 and 30 and 31

EMBASE

1. Child*.tw
2. Exp Child/
3. Adolescen*.tw
4. Exp Adolescent/
5. Student*.tw
6. Exp Students/
7. Youth*.tw
8. Pupil*.tw
9. Schoolchild*.tw
10. (young adj (people or person)).tw
11. teen*.ti,ab.
12. p?diatric*.ti,ab.
13. exp suicide/
14. exp Suicide, Attempted/
15. exp Suicidal ideation/
16. exp suicide attempt/
17. exp suicidal behavior/
18. exp Drug overdose/
19. exp Self-injurious behaviour or exp Self Mutilation/
20. (autoaggress$ or auto aggress$ or automutilat$ or auto mutilat$ or cutt$ or overdose$ or (self adj2 cut$) or selfdestruct$ or self destruct$ or selfharm$ or self harm$ or selfimmolat$ or self immolat$ or selfinflict$ or self inflict$ or selfinjur$ or self injur$ or selfmutilat$ or self mutilat$ or selfpoison$ or self poison$ or suicid$).ti,ab.
21. (expulsion adj5 school*).ti,ab
22. (Suspen* adj5 school*).ti,ab.
23. (manage* adj move*).ti,ab.
24. (Terminat* adj5 school*).ti,ab.
25. (expel* adj5 school*).ti,ab.
26. ((exclusion or exclude*) adj6 school*).ti,ab.
27. ("failure to complete" adj5 (school* or educat*)).ti,ab.
28. ((school* or kindergarten or nursery or education*) adj4 (attend* or non-attend* or refus* or absen* or school phobi* or truan*)).tw.
29. exp school attendance/
30. 1 or 2 or 3 or 4 or 5 or 6 or 7 or 8 or 9 or 10 or 11 or 12
31. 13 or 14 or 15 or 16 or 17 or 18 or 19 or 20
32. 21 or 22 or 23 or 24 or 25 or 26 or 27 or 28 or 29
33. 30 and 31 and 32

Medline

1. child*.tw.
2. exp Child/
3. adolescen*.tw.
4. exp Adolescent/
5. student*.tw.
6. exp Students/
7. youth*.tw.
8. pupil*.tw.
9. schoolchild*.tw.
10. (young adj (people or person)).tw.
11. teen*.ti,ab.
12. p?diatric*.ti,ab.
13. exp suicide/
14. exp Suicide, Attempted/
15. exp Suicidal Ideation/
16. exp Drug Overdose/
17. exp Self-Injurious Behavior/ or exp Self Mutilation/
18. (autoaggress$ or auto aggress$ or automutilat$ or auto mutilat$ or cutt$ or overdose$ or (self adj2 cut$) or selfdestruct$ or self destruct$ or selfharm$ or self harm$ or selfimmolat$ or self immolat$ or selfinflict$ or self inflict$ or selfinjur$ or self injur$ or selfmutilat$ or self mutilat$ or selfpoison$ or self poison$ or suicid$).ti,ab.
19. (expulsion adj5 school*).ti,ab.
20. (Suspen* adj5 school*).ti,ab.
21. (manage* adj move*).ti,ab.
22. Terminat* adj5 school*).ti,ab.
23. (expel* adj5 school*).ti,ab.
24. ((exclusion or exclude*) adj6 school*).ti,ab.
25. ("failure to complete" adj5 (school* or educat*)).ti,ab.
26. ((school* or kindergarten or nursery or education*) adj4 (attend* or non-attend* or refus* or absen* or school phobi* or truan*)).tw.
27. 1 or 2 or 3 or 4 or 5 or 6 or 7 or 8 or 9 or 10 or 11 or 12
28. 13 or 14 or 15 or 16 or 17 or 18
29. 19 or 20 or 21 or 22 or 23 or 24 or 25 or 26
30. 27 and 28 and 29

ERIC

S47	S18 AND S43 AND S46
S46	S19 OR S20 OR S21 OR S22 OR S44 OR S45
S45	AB (autoaggress* OR “auto aggress*” OR automutilat* OR “auto mutilate*” OR cutt* OR overdos* OR (self N2 cut*) OR self-destruct* OR “self destruct*” OR selfharm* OR “self harm*” OR selfimmolat* OR “self immolate*” OR selfinflict* OR “self inflict*” OR selfinjur* OR “self injur*” OR selfmutilat* OR “self mutilate*” OR selfpoison* OR “self poison*” OR suicid*)
S44	AB (autoaggress* OR “auto aggress*” OR automutilat* OR “auto mutilate*” OR cutt* OR overdos* OR (self N2 cut*) OR self-destruct* OR “self destruct*” OR selfharm* OR “self harm*” OR selfimmolat* OR “self immolate*” OR selfinflict* OR “self inflict*” OR selfinjur* OR “self injur*” OR selfmutilat* OR “self mutilate*” OR selfpoison* OR “self poison*” OR suicid*)
S43	S23 OR S24 OR S25 OR S26 OR S27 OR S28 OR S29 OR S30 OR S31 OR S32 OR S33 OR S34 OR S35 OR S36 OR S37 OR S38 OR S39 OR S40 OR S41 OR S42
S42	DE "Withdrawal (Education)"
S41	DE "Attendance" OR DE "Average Daily Attendance" OR DE "College Attendance"
S40	DE "Suspension"
S39	DE "Expulsion"
S38	AB expel*
S37	TI expel*
S36	AB suspension
S35	TI suspension
S34	AB expulsion
S33	TI expulsion
S32	AB "failure to complete"
S31	TI "failure to complete"
S30	AB (exclusion OR exclude*)
S29	TI (exclusion OR exclude*)
S28	AB (terminat* N5 school*)
S27	TI (terminat* N5 school*)
S26	AB (manage* N1 move*)
S25	TI (manage* N1 move*)
S24	AB ((school* OR kindergarten OR nursery OR education*) N4 (attend* OR non-attend* OR refus* OR absen* OR school phobi* OR truan* OR dropout*))

S23	TI ((school* OR kindergarten OR nursery OR education*) N4 (attend* OR non-attend* OR refus* OR absen* OR school phobi* OR truan* OR dropout*))
S22	DE "Self Destructive Behavior"
S21	AB suicid*
S20	TI suicid*
S19	DE "Suicide"
S18	S1 OR S2 OR S3 OR S4 OR S5 OR S6 OR S7 OR S8 OR S9 OR S10 OR S11 OR S12 OR S13 OR S14 OR S15 OR S16 OR S17
S17	AB (young N1 (people OR person))
S16	TI (young N1 (people OR person))
S15	AB schoolchild*
S14	TI schoolchild*
S13	AB pupil*
S12	TI pupil*
S11	AB youth*
S10	TI youth*
S9	DE "Students"
S8	AB student*
S7	TI student*
S6	DE "Adolescents"
S5	AB adolescen*
S4	TI adolescen*
S3	AB child*
S2	TI child*
S1	DE "Child Health" OR DE "Children"

BEI

S49	S20 AND S25 AND S48
S48	S26 OR S27 OR S28 OR S29 OR S30 OR S31 OR S32 OR S33 OR S34 OR S35 OR S36 OR S37 OR S38 OR S39 OR S40 OR S41 OR S42 OR S43 OR S44 OR S45 OR S46 OR S47
S47	DE "SCHOOL dropouts"
S46	DE "COLLEGE attendance"
S45	DE "SCHOOL attendance"
S44	DE "STUDENT suspension"
S43	DE "EXCLUSION from school"

S42	DE "STUDENT expulsion"
S41	AB expel*
S40	TI expel*
S39	AB suspension
S38	TI suspension
S37	AB expulsion
S36	TI expulsion
S35	AB "failure to complete"
S34	TI "failure to complete"
S33	AB (exclusion OR exclude*)
S32	TI (exclusion OR exclude*)
S31	AB (terminat* N5 school*)
S30	TI (terminat* N5 school*)
S29	AB (manage* N1 move*)
S28	TI (manage* N1 move*)
S27	AB ((school* OR kindergarten OR nursery OR education*) N4 (attend* OR non-attend* OR refus* OR absen* OR school phobi* OR truan* OR dropout*))
S26	TI ((school* OR kindergarten OR nursery OR education*) N4 (attend* OR non-attend* OR refus* OR absen* OR school phobi* OR truan* OR dropout*))
S25	S21 OR S22 OR S23 OR S24
S24	AB (autoaggress* OR “auto aggress*” OR automutilat* OR “auto mutilate*” OR cutt* OR overdos* OR (self N2 cut*) OR self-destruct* OR “self destruct*” OR selfharm* OR “self harm*” OR selfimmolat* OR “self immolate*” OR selfinflict* OR “self inflict*” OR selfinjur* OR “self injur*” OR selfmutilat* OR “self mutilate*” OR selfpoison* OR “self poison*” OR suicid*)
S23	AB (autoaggress* OR “auto aggress*” OR automutilat* OR “auto mutilate*” OR cutt* OR overdos* OR (self N2 cut*) OR self-destruct* OR “self destruct*” OR selfharm* OR “self harm*” OR selfimmolat* OR “self immolate*” OR selfinflict* OR “self inflict*” OR selfinjur* OR “self injur*” OR selfmutilat* OR “self mutilate*” OR selfpoison* OR “self poison*” OR suicid*)
S22	AB suicid*
S21	TI suicid*
S20	S1 OR S2 OR S3 OR S4 OR S5 OR S6 OR S7 OR S8 OR S9 OR S10 OR S11 OR S12 OR S13 OR S14 OR S15 OR S16 OR S17 OR S18 OR S19
S19	AB (young N1 (people OR person))
S18	TI (young N1 (people OR person))
S17	AB schoolchild*

S16	TI schoolchild*
S15	AB pupil*
S14	TI pupil*
S13	AB youth*
S12	TI youth*
S11	DE "STUDENTS"
S10	AB student*
S9	TI student*
S8	DE "ADOLESCENT psychology"
S7	(DE "TEENAGERS") OR (DE "ADOLESCENCE")
S6	AB adolescen*
S5	TI adolescen*
S4	AB child*
S3	TI child*
S2	(DE "CHILD psychology") OR (DE "CHILD psychiatry")
S1	(DE "CHILDREN") OR (DE "CHILDREN -- Health")
