# Supplementary material for: Poor school attendance and exclusion: a systematic review protocol on educational risk factors for self-harm and suicidal behaviours
Source: BMJ Open. 2018 Dec 14;8(12):e023953. doi: 10.1136/bmjopen-2018-023953 (PMC6303662; doi:10.1136/bmjopen-2018-023953)
Supplement: Supplementary file 3 [file bmjopen-2018-023953supp003.pdf]

**Data Extraction**

Reference:

Self-harm variable
Type and duration:

Binary/other:

Method of ascertainment:

Attendance/exclusion variable
Type and duration:

Binary/other:

Method of ascertainment:

Covariates:

N subjects (or cases/controls):

Characteristics of cohort:

Country:

Age (mean/median and range):

Study design:

Time period of data collection:

Odds ratio(s) or other effect estimate(s):

Confidence interval(s):

P Value(s):

Notes about results:

**For quality assessment (See risk of bias tools for more information)**

Cross sectional:
Representativeness of sample (0-1):
Sample size (0-1):
Non-respondents (0-1):
Ascertainment of attendance/exclusion variable (0-1):
Comparability (0-2):
Ascertainment of self-harm variable (0-1):
Statistical reporting (0-1):
Clarity of variable definitions (0-2):

Case control:
Case definition (0-1):
Representativeness of cases (0-1):
Selection of controls (0-1):
Definition of controls (0-1):
Comparability (0-2):
Ascertainment of attendance/exclusion variable (0-1):
Same ascertainment method for cases and controls (0-1):
Non-response rate (0-1):
Sample size (0-1):
Statistical reporting (0-1):
Clarity of variable definitions (0-2):

Cohort:
Representativeness of exposed cohort (0-1):
Selection of non-exposed cohort (0-1):
Definition of controls (0-1):
Ascertainment of attendance/exclusion variable (0-1):
Outcome not present at start (0-1):
Comparability (0-2):
Ascertainment of self-harm variable (0-1):
Follow-up long enough (0-1):
Adequacy of follow-up (0-1):
Sample size (0-1):
Statistical reporting (0-1):
Clarity of variable definitions (0-2):
