# Supplementary material for: Poor school attendance and exclusion: a systematic review protocol on educational risk factors for self-harm and suicidal behaviours
Source: BMJ Open. 2018 Dec 14;8(12):e023953. doi: 10.1136/bmjopen-2018-023953 (PMC6303662; doi:10.1136/bmjopen-2018-023953)
Supplement: Supplementary file 4 [file bmjopen-2018-023953supp004.pdf]

# Adapted Newcastle-Ottawa Quality Assessment Scales

## Cohort Studies

Note: A study can be awarded a maximum of one point for each numbered item with the Selection and Exposure categories. A maximum of two points can be given for Comparability.

**Selection (maximum 4)**

1. Representativeness of the cohort
    a. Truly representative of the average in the target population (random sample or whole population)*
    b. Somewhat representative of the average in the target population (purposive sampling of representative schools or evidence that the sample is representative of the source population)*
    c. Selected group of users
    d. No description of the derivation of the cohort
2. Selection of the non-exposed cohort
    a. Drawn from the same community as the exposed cohort *
    b. Drawn from a different source
    c. No description of the derivation of the non-exposed cohort
3. Ascertainment of exposure (absence or exclusion)
    a. School administrative records*
    b. Reported by school staff*
    c. Other collateral or self-report
    d. No description
4. Demonstration that outcome of interest was accounted for or not present at start of study
    a. Yes *
    b. No

**Comparability (maximum 2)**

1. Comparability of cohorts on the basis of the design or analysis
    a. Study controls for age and gender (or analysis separated by gender) *
    b. Study controls for any additional factor *

**Outcome (maximum 3)**

1. Ascertainment of outcome (self-harm or suicidality)
    a. Structured interview or written self-report*
    b. Clinical record*
    c. Reported by school or collateral
    d. No description
2. Was follow-up long enough for outcomes to occur?
    a. Yes (>1 year) *
    b. No
3. Adequacy of follow-up of cohorts
    a. Complete follow-up – all subjects accounted for *
    b. Subjects lost to follow-up unlikely to introduce bias – small number lost (<20%) or attrition described and accounted for in analysis *
    c. Follow up rate not adequate and no description of those lost

d. No statement

----

Note: A study can be awarded a maximum of one point for each numbered item for sample size and statistical test. A maximum of two points can be given for clear variables.

Additional parameters: Not in NOS, taken from cross sectional NOS:

1. Sample size (Maximum 1)
    a. Justified and satisfactory *
    b. Adequately powered to detect a difference (at least 10 events per variable in multivariate analyses)*
    c. Not justified

2. Statistical test (Maximum 1)
    a. The statistical test used to analyse the data is clearly described and appropriate, and the measurement of the association is presented as either an OR, CI and P value or a beta coefficient, SE and P value*
    b. The statistical test is not appropriate, not described or incomplete

Further additional parameter:

1. Clear variables (Maximum 2)
    a. Both variables clearly defined including time period for measurement (2)
    b. One or other variable clearly defined including time period for measurement (1)
    c. Neither variable clearly defined including time period for measurement (0)

## Case-Control Studies

Note: A study can be awarded a maximum of one point for each numbered item with the Selection and Exposure categories. A maximum of two points can be given for Comparability.

**Selection (maximum 4)**

5. Is the case definition adequate? (self-harm or suicidality)
    a. Structured interview or written self-report*
    b. Clinical record*
    c. Reported by school or collateral
    d. No description
6. Representativeness of the cases
    a. Consecutive or obviously representative series of cases *
    b. Potential for selection biases or not stated
7. Selection of controls
    a. Community controls from source population*
    b. Clinical controls if clinical source population*
    c. Not extracted from same source population
    d. No description
8. Definition of controls

    a. No history of outcome (NB if cases have new (not necessarily first) occurrence of outcome, controls with previous occurrences should not be excluded) *
    b. No description of source

**Comparability (maximum 2)**

2. <u>Comparability of cases and controls on the basis of the design or analysis</u>
    a. Study controls for age and gender (or analysis separated by gender)*
    b. Study controls for any additional factor *

**Exposure (maximum 3)**

4. <u>Ascertainment of exposure (absence or exclusion)</u>
    a. School administrative records*
    b. Reported by school staff*
    c. Other collateral or self-report
    d. No description
5. <u>Same method of ascertainment for cases and controls</u>
    a. Yes *
    b. No
6. <u>Non-response rate</u>
    a. Same rate for both groups *
    b. Non-response or missing values should be <20% and accounted for in analysis*
    c. Non respondents described
    d. Rate different and no designation

---

Note: A study can be awarded a maximum of one point for each numbered item for sample size and statistical test. A maximum of two points can be given for clear variables.

Additional parameters: Not in NOS, taken from cross sectional NOS:

3. <u>Sample size (Maximum 1)</u>
    a. Justified and satisfactory *
    b. Adequately powered to detect a difference (at least 10 events per variable in multivariate analyses)*
    c. Not justified

4. <u>Statistical test (Maximum 1)</u>
    a. The statistical test used to analyse the data is clearly described and appropriate, and the measurement of the association is presented as either an OR, CI and P value or a beta coefficient, SE and P value*
    b. The statistical test is not appropriate, not described or incomplete

Further additional parameter:

2. <u>Clear variables (Maximum 2)</u>
    a. Both variables clearly defined including time period for measurement (2)
    b. One or other variable clearly defined including time period for measurement (1)
    c. Neither variable clearly defined including time period for measurement (0)

## Cross-Sectional Studies

Note: A study can be awarded a maximum of one point for each numbered item with the Selection and Exposure categories. A maximum of two points can be given for Comparability.

**Selection (maximum 4)**

9. Representativeness of the sample
    a. Truly representative of the average in the target population (random sample or whole population) *
    b. Somewhat representative of the average in the target population (purposive sampling of representative schools or evidence that the sample is representative of the source population) *
    c. Selected group of users
    d. No description of the sampling strategy
10. Sample size
    a. Justified and satisfactory *
    b. Adequately powered to detect a difference (at least 10 events per variable in multivariate analyses)*
    c. Not justified
11. Non-respondents
    a. Comparability between respondents and non-respondents characteristics is established, and the response rate is satisfactory (>60%)*
    b. The response rate is unsatisfactory, or the comparability between respondents and non-respondents is unsatisfactory
    c. No description of the response rate or the characteristics of the responders and non-responders
12. Ascertainment of the exposure (absence or exclusion)
    a. School administrative records*
    b. Reported by school staff*
    c. Other collateral or self-report
    d. No description

**Comparability (maximum 2)**

3. The subjects in different outcome groups are comparable, based on the study design or analysis. Confounding factors are controlled
    a. Study controls for age and gender (or analysis separated by gender)*
    b. Study controls for any additional factor *

**Outcome (maximum 2)**

7. Assessment of the outcome (self-harm or suicidality)
    a. Structured interview or written self-report*
    b. Clinical record*
    c. Reported by school or collateral
    d. No description

8. Statistical test

a. The statistical test used to analyse the data is clearly described and appropriate, and the measurement of the association is presented as either an OR, CI and P value or a beta coefficient, SE and P value*
b. The statistical test is not appropriate, not described or incomplete

---

Additional parameter not in NOS:

3. <u>Clear variables (Maximum 2)</u>
    a. Both variables clearly defined including time period for measurement (2)
    b. One or other variable clearly defined including time period for measurement (1)
    c. Neither variable clearly defined including time period for measurement (0)
