# Supplementary material for: Poor school attendance and exclusion: a systematic review protocol on educational risk factors for self-harm and suicidal behaviours
Source: BMJ Open. 2018 Dec 14;8(12):e023953. doi: 10.1136/bmjopen-2018-023953 (PMC6303662; doi:10.1136/bmjopen-2018-023953)
Supplement: Author's manuscript [file bmjopen-2018-023953.draft_revisions.pdf]

BMJ Open is committed to open peer review. As part of this commitment we make the peer review history of every article we publish publicly available.

When an article is published we post the peer reviewers' comments and the authors' responses online. We also post the versions of the paper that were used during peer review. These are the versions that the peer review comments apply to.

The versions of the paper that follow are the versions that were submitted during the peer review process. They are not the versions of record or the final published versions. They should not be cited or distributed as the published version of this manuscript.

BMJ Open is an open access journal and the full, final, typeset and author-corrected version of record of the manuscript is available on our site with no access controls, subscription charges or pay-per-view fees (http://bmjopen.bmj.com).

If you have any questions on BMJ Open's open peer review process please email info.bmjopen@bmj.com

BMJ Open

Poor school attendance and exclusion: a systematic review protocol on educational risk factors for self-harm and suicidal behaviours

Journal:	*BMJ Open*
Manuscript ID	bmjopen-2018-023953
Article Type:	Protocol
Date Submitted by the Author:	02-May-2018
Complete List of Authors:	Epstein, Sophie; South London and Maudsley NHS Foundation Trust, NIHR Maudsley Biomedical Research Centre; King's College London, Department of Child and Adolescent Psychiatry<br>Roberts , Emmert; King's College London, National Addiction Centre & Department of Psychological Medicine; South London and Maudsley NHS Foundation Trust<br>Sedgwick, Rosemary; South London and Maudsley NHS Foundation Trust; King's College London, Department of Child and Adolescent Psychiatry<br>Finning, Katie; University of Exeter Medical School<br>Ford, Tamsin; University of Exeter Medical School<br>Dutta, Rina; Kings College London, Department of Psychological Medicine ; South London and Maudsley NHS Foundation Trust, NIHR Maudsley Biomedical Research Centre<br>Downs, Johnny; Kings College London, Department of Child and Adolescent Psychiatry ; South London and Maudsley NHS Foundation Trust, NIHR Maudsley Biomedical Research Centre
Keywords:	Child & adolescent psychiatry < PSYCHIATRY, Suicide & self-harm < PSYCHIATRY, School mental health, Systematic review protocol, Epidemiology < TROPICAL MEDICINE

SCHOLARONE™
Manuscripts

For peer review only - http://bmjopen.bmj.com/site/about/guidelines.xhtml

Poor school attendance and exclusion: a systematic review protocol on educational risk factors for self-harm and suicidal behaviours

Sophie Epstein[1,2,3]*, Emmert Roberts[2,4,5], Rosemary Sedgwick[2,3], Katie Finning[6], Tamsin Ford[6], Rina Dutta[1,2,5], Johnny Downs[1,2,3]

1. NIHR Maudsley Biomedical Research Centre, South London and Maudsley NHS Foundation Trust
2. South London and Maudsley NHS Foundation Trust
3. Department of Child and Adolescent Psychiatry, King's College London
4. National Addiction Centre, King's College London
5. Department of Psychological Medicine, King's College London
6. University of Exeter Medical School

*Corresponding author

Abstract

Introduction

Schools have an important role in recognising and preventing self-harm and suicidal behaviour in their students, however little is known about which educational factors are associated with heightened risk. We will systematically review the existing evidence on two key educational performance indicators that are routinely collected by school administrative systems: school attendance and exclusion. We will investigate their association with self-harm and suicidal behaviour in school-age children and adolescents. Knowledge of this association could help to inform suicide prevention strategies at clinical, school and population level.

Methods and analysis

We will conduct a systematic search of Medline, EMBASE, PsycINFO, British Education Index and Education Resources Information Centre (ERIC) from 01/01/1990, and conduct a manual search for additional references. We aim to identify studies that explore the association between poor school attendance or exclusion and self-harm or suicidal behaviours in school-age children and adolescents. Two independent reviewers will screen titles, abstracts and full text documents and independently extract relevant data for analysis. Study quality will be assessed using a modified Newcastle-Ottawa Scale. A descriptive analysis will be performed, and where appropriate, results will be combined in meta-analyses.

Ethics and dissemination

This is a systematic review of published literature, and therefore ethical approval will not be sought. We will publish reports in health and education journals, present our work at conferences focused on school mental health and communicate our findings to practitioners and managers in public health, education and child mental health.

Registration details

The review is registered on PROSPERO. ID CRD42018088608

For peer review only

Strengths and limitations of this study

1. This will be the first systematic review to investigate poor school attendance and exclusion as risk factors for self-harm and suicidal behaviour in school-age children and adolescents.

2. The methods involve a systematic search of databases and other sources from both health and education fields, including comprehensive forward and backward citation searching and contacting key authors in the field.

3. The wide inclusion criteria mean that this review will serve as a broad overview of the questions of interest and will allow identification of particular gaps in the existing evidence base.

4. The use of wide inclusion criteria may also create challenges in synthesising the evidence as constructs described in each study may not be comparable.

5. This systematic review does not include non-peer reviewed literature and only includes studies published in the English language and therefore may not report an entirely comprehensive view of the literature on this topic.

Introduction

Self-harm and suicidal behaviour in children and adolescents are important public health issues. International community-based studies have reported a prevalence of self-harm in adolescents of approximately 10%.[1] Suicide is the second most common cause of death worldwide for the 10-24 age group [2] and national studies in several countries have found that on average between 1 and 10% of adolescents report having attempted suicide during their lifetime.[3-7] Self-harm and attempted suicide are amongst the strongest predictors of completed suicide [8 9] and are strongly associated with mental disorders including depression and anxiety.[6]

In the UK, the focus of current child and adolescent mental health policy is on developing closer collaborations between the health and education sectors and supporting schools to develop capacity to play a greater role in supporting and promoting pupils' mental health. [10] This includes a role in early identification of those at risk of mental health difficulties or risky behaviours including self-harm. Currently, many school-based suicide prevention programmes exist, but most constitute universal prevention rather than using strategies that target particular at-risk groups.[11] In the UK, it is unclear how broadly such interventions are implemented or how at-risk groups are targeted (Evans, Parker, Russell et al, Adolescent self-harm prevention and intervention in secondary schools: A survey of staff in England and Wales, In Submission).

Any policy development needs to be informed by robust evidence on the factors that could help to identify those children and adolescents at greater risk of self-harm or suicidal behaviours. A number of risk factors for self-harm and suicidal behaviour in children and adolescents have been identified, which include both psychological factors such as mental disorders, drug and alcohol misuse; personality characteristics such as impulsivity; as well as socio-demographic factors such as female sex for self-harm, and male sex for completed suicide, low socio-economic status, adverse childhood experiences, family discord and bullying.[1 12 13] Risk profiles are also found to vary between subgroups when stratified by certain socio-demographic factors such as gender, age and ethnicity.[1 3 14]

A small number of studies have reported an association between suicidal phenomena and poor school attendance, actual and perceived educational attainment, poor school

For peer review only - http://bmjopen.bmj.com/site/about/guidelines.xhtml

connectedness and experiences, and negative attitudes towards school.[1 12 15-20] There is also evidence of higher rates of suicide attempts among young adults with a history of poor school performance [21 22] but a lower risk amongst those who attended schools with a lower average academic performance.[22]

Beyond this however, educational factors, which may be particularly pertinent in this age-group, are yet to be explored in any detail. This is surprising, given that data on these factors are routinely collected by education systems in many high income countries.

Although many systematic reviews that explore risk factors for self-harm and suicidal behaviour among children and adolescents have been published, we have not been able to identify any which specifically report on educational risk factors, and none which attempt to comprehensively identify the literature that explores poor school attendance and school exclusion as possible risk factors for self-harm and suicidal behaviour.

In this systematic review, poor school attendance and exclusion from school are hypothesised to be two potentially important risk factors for self-harm and suicidal behaviours in school-age populations. We seek to answer the question; are poor attendance or exclusion from school associated with self-harm and/or suicidal behaviour in school-age children and adolescents? We also aim to explore whether this association persists after accounting for other potentially confounding socio-demographic risk factors for self-harm and suicidal behaviour such as age, gender and ethnicity.

Methods:

This protocol follows the Preferred Reporting Items for Systematic review and Meta-Analysis Protocols (PRISMA-P)[23] guidelines and is registered on PROSPERO, an international register of systematic reviews ID=CRD42018088608.[24] Any changes to the protocol will be recorded on PROSPERO.

Eligibility criteria:

Population

Eligible participants will be those aged 4-18 and attending school at the point of enrolment into the study or where poor attendance or exclusion from school and at least a proportion of the self-harm or suicidal behaviour are measured during this age range. The reason for this choice of age range is that it constitutes the school age population in most countries.

Types of studies

Any observational studies (cohort, longitudinal, case control, cross sectional) published from 1990 onwards will be included. Qualitative studies, case reports, intervention studies and comments/editorials will be excluded. Studies conducted in any country will be included, provided they are published in English. The reason for exclusion of older studies, is that due to the changing nature of educational systems and social contexts within which self-harm occurs, such studies are less relevant to the present day. Only peer reviewed papers will be included, and only those where the full text of the study is available. Grey literature will not be included.

Exposure and outcome variables

Any study that investigates a possible association between poor school attendance or school exclusion and self-harm or suicidal behaviour will be included. Studies will only be

For peer review only - http://bmjopen.bmj.com/site/about/guidelines.xhtml

included if there is a comparison group. In order to be included, a study will need to report poor school attendance or exclusion as an exposure and self-harm or suicidal behaviour as an outcome. Exceptions to this rule are cross sectional studies that consider self-harm or suicidal behaviour as an exposure and poor attendance or exclusion as an outcome, and which report results for univariate analyses.

The existing literature on self-harm and suicidal behaviour is extremely heterogeneous in terms of the terminology used. We will therefore consider a wide definition to encompass this variation, which will include any form of self-harm or thoughts about self-harm, and any form of suicidal behaviour including suicidal thoughts or ideation, plans, attempts, or completed suicide. The data on this variable can be ascertained by any method including written self-reports, interviews, collateral reports or clinical or other secure records.

In terms of poor school attendance and exclusion, again a broad definition will be considered as terminology used in the international literature varies widely. Acceptable variables will include any form of exclusion or suspension from school, temporary or permanent, over any period of time. Any authorised or unauthorised non-attendance at school (amongst pupils enrolled in school) including school refusal, school phobia, truancy or long term absence due to ill health will be included. Studies using ‘school dropout’ as an exposure of interest will be excluded as this considered a different construct to poor school attendance. Additionally, those studies that consider attendance in relation to whether the child is or is not enrolled at school, will be excluded. Attendance in relation to being on school roll or not taps a different set of issues and is of greatest relevance to populations in low and middle income countries where some children do not attend school for financial reasons or due to lack of education provision. Any method of data collection will be acceptable, including self or collateral reports or secure records.

Search methods:
The following health and education databases will be searched as follows:
- Medline (OvidSP platform)
- Embase (OvidSP platform)
- PsycINFO (OvidSP platform)
- British Education Index (EBSCO platform)
- Education Resources Information Centre (EBSCO platform)

Databases will be searched from 01/01/1990 until present. The search strategy is described below.

Citation lists of included studies will then be checked for other relevant studies (backward citation searching), and forward citation searching will be conducted by systematically looking through lists of papers which have subsequently cited the included studies. Where possible, Web of Science will be used for this purpose, and in situations where the paper is not listed on Web of Science, Google Scholar will be used. We will also look through the reference lists of existing systematic reviews on similar topics to identify any further relevant papers. If any key journals are identified during the initial searches, we will hand search these journals to look for additional relevant papers.

Once a final list of papers has been generated using the above methods, experts in the field will be contacted to identify any additional papers.

For peer review only

Search strategy for electronic databases:

The search strategies have been developed to include both keywords and thesaurus terms relevant to each respective database. For keyword searching, truncation and wildcards are used as necessary to allow for linguistic variations. Full search strategies can be found in Supplementary File 1. The included terms refer to the following concepts:

- Population (e.g. child, adolescent)
- Attendance/exclusion (e.g. absence, truancy, exclusion)
- Self-harm/suicidal behaviour (e.g. suicidal, self-harm)

Due to the wide range of possible terms to describe self-harm, the list of terms used for this part of the search strategy has been taken from a recent review on suicide and self-harm published in the British Journal of Psychiatry.[25] Similarly, the terms for poor school attendance were taken from a related systematic review protocol which was developed in consultation with an information specialist as well as experts in the fields of child mental health and education.[26]

Selection of references:

Titles and abstracts generated from the searches of electronic databases will be exported to Endnote X8 software and duplicates removed. The references will then be screened for eligibility by two independent reviewers. Any uncertainties will be discussed between the two reviewers (SE and RS). Any discrepancies between the decisions of the two reviewers will be initially discussed between them, and if a consensus cannot be reached, a third reviewer (JD) will adjudicate. Included full texts will be obtained and screened by two reviewers and again any disagreement will be discussed with a third reviewer.

Data extraction

Data will be extracted by two independent reviewers (SE and ER) according to an agreed data extraction form (Supplementary File 2). Data to be extracted will include: Definitions of exposure and outcome variables and methods of ascertainment, covariates included in analysis, characteristics of the cohort including age and gender balance of participants, country, study design, relevant results (including effect estimates, confidence intervals, and p-values, where reported) and information for the assessment of study quality/risk of bias. Where two studies report data from the same cohort, the larger sample, or if the same sample, the study with the longest follow up will be used to ensure participants are not double counted.

Quality assessment

Risk of bias within the included studies will be assessed using modified Newcastle-Ottawa Scales (NOS) for assessment of risk of bias in observational studies.[27] The modified scales can be found in Supplementary File 3. This will be carried out by two independent reviewers (SE and ER). As there is no original version of the NOS for cross-sectional studies, an adapted version used in previously published literature, will be used.[28] Some items on the NOS scales have been adapted for relevance to this research question, in particular the items relating to acceptable methods of ascertainment of the exposure and outcome variables. Further key quality parameters will be assessed in addition to the NOS items as follows: appropriate sample size, appropriate statistical tests and clarity of reporting of exposure and outcome variables. Any discrepancies will be discussed between the two reviewers, and if a consensus cannot be reached, a third reviewer will adjudicate.

For peer review only - http://bmjopen.bmj.com/site/about/guidelines.xhtml

Analysis

Included studies will be described according to their respective characteristics in the text and in tables, and the results of the studies will be discussed in a narrative synthesis, bringing together studies according to particular outcomes, exposures or type of participant where possible. Results will be standardised where possible by reporting or calculating odds ratios. If any two or more studies use sufficiently similar methodology, exposure and outcome variables, meta-analyses will be conducted. A random effects model will be used, due to the likelihood of considerable heterogeneity between studies, and we will present a summary effect with an associated 95% confidence interval and P value. The level of statistical heterogeneity will be determined using the I^2 statistic. Where there is a large enough number of studies within a meta-analysis, publication bias will be assessed using funnel plots and Eggers test. An assessment of the strength of the evidence per outcome will be conducted using the Grading of Recommendations Assessment, Development and Evaluation (GRADE) approach adapted for non-randomised studies. Where possible, results stratified by gender will be presented and if the data within studies allows, we will also report subgroup analyses by age and ethnicity.

Findings from all studies will be reported and included in the descriptive analysis regardless of their risk of bias. The quality of included studies, as assessed by the NOS as described above, will be taken into consideration in the synthesis and analysis of the results, in terms of the strength of the evidence within each group of studies considered. We will conduct a sensitivity analysis removing any papers which score below 60% on the quality assessment scales and report a synthesis of the evidence from the remaining studies to assess whether this has an impact on the overall effect.

Discussion

This systematic review aims to bring together international evidence on the association between important educational risk factors and a common and important outcome of self-harm and suicidal behaviour in children and adolescents. We are aware that all of these variables constitute broad constructs and the terminology used to describe these constructs varies widely in different contexts. It is therefore likely that there will be challenges involved in synthesising data on this this topic.

An additional challenge is that studies may report poor school attendance and exclusion among many other variables considered as possible risk factors for self-harm and suicidal behaviour. It is possible therefore that terms referring to these particular exposures are not referenced in the titles or abstracts of the studies. We will minimise the risk of missing relevant studies by employing a range of search methods including consultation with experts to identify any additional papers.

Despite these challenges, this systematic review will be the first to specifically address the question of whether poor school attendance and exclusion are related to self-harm and suicidal behaviour in young people. We will rigorously assess the quality and strength of the evidence, which could generate further research questions, and could ultimately help to inform suicide prevention strategies at clinical, school or population level.

For peer review only - http://bmjopen.bmj.com/site/about/guidelines.xhtml

In the UK and many other high income countries, data on school attendance and exclusions are routinely collected for every child in the state education system. Additionally, compared to many other parameters collected, such as socio-economic status and special educational needs, these are relatively objective and standardised measures, which could be used to identify those at higher risk of adverse outcomes, including self-harm and suicidal behaviour. With the opportunity for schools to play an important role in the prevention of self-harm and suicide,[10] it is crucial to know whether these routinely collected factors can be used as predictors of those at higher risk. The presence or absence of an association has important policy implications in terms of the design and implementation of targeted school-based suicide prevention interventions. If evidence on this association (or other important factors which may provide an alternative explanation for this association) is found to be lacking, an urgent need for further research will be highlighted.

Word count: 2646

Ethics and dissemination

As this is a systematic review of published literature, ethical approval will not be sought. We will publish the protocol and our findings in peer-reviewed journals aimed at school and health commissioners, practitioners and managers. We will present our work at the growing numbers of national and international meetings focused on school mental health. This work will be used to inform our ongoing work with the Department for Education to examine educational risk factor for child and adolescent mental health outcomes, including secondary analyses of the National Pupil Database using linked health and school data.

Author contributions

SE (Guarantor) and JD conceived and designed the review with input from all other authors. All authors contributed to critically revising the manuscript and approved the final version.

Funding statement

SE has been employed as a National Institute of Health Research (NIHR) Academic Clinical Fellow and currently receives salary support from an MQ Data Science Award and from the Psychiatry Research Trust. ER is funded by a Medical Research Council (MRC) Addiction Research Clinical Training Fellowship. RS is employed as an NIHR Academic Clinical Fellow. KF’s PhD is funded by the University of Exeter, via the MYRIAD study (Wellcome Trust 107496/Z/15/Z). RD is funded by a Clinician Scientist Fellowship (research project e-HOST-IT) from the Health Foundation in partnership with the Academy of Medical Sciences. JD received support via a MRC Clinical Research Training Fellowship (MR/L017105/1), Psychiatry Research Trust Peggy Pollak Research Fellowship in Developmental Psychiatry and from the NIHR Biomedical Research Centre at South London and Maudsley NHS Foundation Trust and King’s College London. This paper represents independent research part funded by the National Institute for Health Research (NIHR) Biomedical Research Centre at South London and Maudsley NHS Foundation Trust and King’s College London. The views expressed are those of the authors and not necessarily those of the NHS, the NIHR or the Department of Health and Social Care.

Competing interests declaration

The authors have no competing interests

Correspondence

For peer review only

Dr Sophie Epstein, NIHR Maudsley Biomedical Research Centre Nucleus, PO Box 005, De Crespigny Park, Denmark Hill, London SE5 8AF, United Kingdom.
Email: Sophie.epstein@kcl.ac.uk

Email addresses of other authors
Emmert Roberts: Emmert.roberts@kcl.ac.uk
Rosemary Sedgwick: Rosemary.sedgwick@slam.nhs.uk
Katie Finning: K.Finning@exeter.ac.uk
Tamsin Ford: T.J.Ford@exeter.ac.uk
Rina Dutta: Rina.dutta@kcl.ac.uk
Johnny Downs Johnny.downs@kcl.ac.uk

Supplementary File 1: Search Strategies

Supplementary File 2: Data extraction form

Supplementary File 3: Quality assessment tools

Supplementary File 4: PRISMA-P checklist

REFERENCES

1. Hawton K, Saunders KE, O'Connor RC. Self-harm and suicide in adolescents. *Lancet* 2012;379(9834):2373-82. doi: 10.1016/s0140-6736(12)60322-5 [published Online First: 2012/06/26]
2. Patton GC, Coffey C, Sawyer SM, et al. Global patterns of mortality in young people: a systematic analysis of population health data. *Lancet (London, England)* 2009;374(9693):881-92. doi: 10.1016/s0140-6736(09)60741-8 [published Online First: 2009/09/15]
3. Nock MK, Green JG, Hwang I, et al. Prevalence, correlates, and treatment of lifetime suicidal behavior among adolescents: results from the National Comorbidity Survey Replication Adolescent Supplement. *JAMA Psychiatry* 2013;70(3):300-10. doi: 10.1001/2013.jamapsychiatry.55 [published Online First: 2013/01/11]
4. Hargus E, Hawton K, Rodham K. Distinguishing Between Subgroups of Adolescents Who Self-Harm. *Suicide and Life-Threatening Behavior* 2009;39(5):518-37. doi: 10.1521/suli.2009.39.5.518
5. Madge N, Hewitt A, Hawton K, et al. Deliberate self-harm within an international community sample of young people: comparative findings from the Child & Adolescent Self-harm in Europe (CASE) Study. *Journal of child psychology and psychiatry, and allied disciplines* 2008;49(6):667-77. doi: 10.1111/j.1469-7610.2008.01879.x [published Online First: 2008/03/18]
6. Moran P, Coffey C, Romaniuk H, et al. The natural history of self-harm from adolescence to young adulthood: a population-based cohort study. *The Lancet* 2012;379(9812):236-43. doi: 10.1016/S0140-6736(11)61141-0
7. Kokkevi A, Rotsika V, Arapaki A, et al. Adolescents' self-reported suicide attempts, self-harm thoughts and their correlates across 17 European countries. *Journal of child*

For peer review only

psychology and psychiatry, and allied disciplines 2012;53(4):381-9. doi: 10.1111/j.1469-7610.2011.02457.x [published Online First: 2011/09/08]
8. Hawton K, Harriss L. Deliberate self-harm in young people: characteristics and subsequent mortality in a 20-year cohort of patients presenting to hospital. *The Journal of clinical psychiatry* 2007;68(10):1574-83. [published Online First: 2007/10/27]
9. Joiner TE, Jr., Conwell Y, Fitzpatrick KK, et al. Four studies on how past and current suicidality relate even when "everything but the kitchen sink" is covaried. *Journal of abnormal psychology* 2005;114(2):291-303. doi: 10.1037/0021-843x.114.2.291 [published Online First: 2005/05/05]
10. DoH D. Transforming Children and Young People's Mental Health Provision: a Green Paper. 2017
11. Katz C, Bolton SL, Katz LY, et al. A systematic review of school-based suicide prevention programs. *Depression and anxiety* 2013;30(10):1030-45. doi: 10.1002/da.22114 [published Online First: 2013/05/08]
12. Evans E, Hawton K, Rodham K. Factors associated with suicidal phenomena in adolescents: a systematic review of population-based studies. *Clinical psychology review* 2004;24(8):957-79. doi: 10.1016/j.cpr.2004.04.005 [published Online First: 2004/11/10]
13. Steele M, Doey T. Suicidal Behaviour in Children and Adolescents. Part 1: Etiology and Risk Factors *The Canadian Journal of Psychiatry* 2007;52(6 Supplement 1):21S-33S.
14. Hawton K, Rodham K, Evans E, et al. Deliberate self harm in adolescents: self report survey in schools in England. *BMJ* 2002;325(7374):1207-11. doi: 10.1136/bmj.325.7374.1207
15. Young R, Sweeting H, Ellaway A. Do schools differ in suicide risk? The influence of school and neighbourhood on attempted suicide, suicidal ideation and self-harm among secondary school pupils. *BMC public health* 2011;11:874.
16. Stallard P, Spears M, Montgomery AA, et al. Self-harm in young adolescents (12-16 years): Onset and short-term continuation in a community sample. *BMC Psychiatry* 2013;13:no pagination.
17. Kidger J, Heron J, Leon DA, et al. Self-reported school experience as a predictor of self-harm during adolescence: a prospective cohort study in the South West of England (ALSPAC). *J Affect Disord* 2015;173:163-9. doi: 10.1016/j.jad.2014.11.003 [published Online First: 2014/12/03]
18. Richardson AS, Bergen HA, Martin G, et al. Perceived academic performance as an indicator of risk of attempted suicide in young adolescents. *Arch Suicide Res* 2005;9(2):163-76. doi: 10.1080/13811110590904016 [published Online First: 2005/07/16]
19. Orozco R, Benjet C, Borges G, et al. Association between attempted suicide and academic performance indicators among middle and high school students in Mexico: results from a national survey. *Child and Adolescent Psychiatry and Mental Health* 2018;12:9. doi: 10.1186/s13034-018-0215-6
20. Lee MT, Wong BP, Chow BW, et al. Predictors of suicide ideation and depression in Hong Kong adolescents: perceptions of academic and family climates. *Suicide Life Threat Behav* 2006;36(1):82-96. [published Online First: 2006/05/09]
21. Kosidou K, Dalman C, Fredlund P, et al. School performance and the risk of suicide attempts in young adults: a longitudinal population-based study. *Psychological medicine* 2014;44(6):1235-43. doi: 10.1017/s0033291713001852 [published Online First: 2013/07/26]

For peer review only

22. Jablonska B, Ostberg V, Hjern A, et al. School effects on risk of non-fatal suicidal behaviour: a national multilevel cohort study. *Soc Psychiatry Psychiatr Epidemiol* 2014;49(4):609-18. doi: 10.1007/s00127-013-0782-z [published Online First: 2013/10/26]
23. Moher D, Shamseer L, Clarke M, et al. Preferred reporting items for systematic review and meta-analysis protocols (PRISMA-P) 2015 statement. *Systematic Reviews* 2015;4(1):1. doi: 10.1186/2046-4053-4-1
24. Booth A, Clarke M, Dooley G, et al. The nuts and bolts of PROSPERO: an international prospective register of systematic reviews. *Systematic Reviews* 2012;1(1):2. doi: 10.1186/2046-4053-1-2
25. Chan MKY, Bhatti H, Meader N, et al. Predicting suicide following self-harm: systematic review of risk factors and risk scales. *The British Journal of Psychiatry* 2016 doi: 10.1192/bjp.bp.115.170050
26. Finning K, Moore D, Ukoumunne OC, et al. The association between child and adolescent emotional disorder and poor attendance at school: a systematic review protocol. *Systematic reviews* 2017;6(1):121. doi: 10.1186/s13643-017-0523-6 [published Online First: 2017/07/01]
27. Wells GA SB, O'Connell D, Peterson J, Welch V, Losos M, Tugwell P. The Newcastle-Ottawa Scale (NOS) for assessing the quality of nonrandomised studies in meta-analyses. *The Ottawa Hospital Research Institute* 2004
28. Herzog R, Alvarez-Pasquin MJ, Diaz C, et al. Are healthcare workers' intentions to vaccinate related to their knowledge, beliefs and attitudes? A systematic review. *BMC public health* 2013;13:154. doi: 10.1186/1471-2458-13-154 [published Online First: 2013/02/21]

For peer review only

Search Strategies

PsycINFO

1. Child*.tw
2. Adolescen*.tw
3. Student*.tw
4. Exp Students/
5. Youth*.tw
6. Pupil*.tw
7. Schoolchild*.tw
8. (young adj (people or person).tw
9. teen*.tw
10. p?diatric.tw
11. exp Elementary School Students /
12. exp Suicide/
13. exp Suicide, Attempted/
14. exp Suicidal Ideation/
15. exp Drug Overdose/
16. exp Self-injurious Behaviour or exp Self Mutilation/
17. (autoaggress* or auto aggress* or automutilat* or cutt* or overdose* or (self adj2 cut*) or selfdestruct* or self destruct* or selfharm* or self harm* or selfimmolat* or self immolat* or selfinflict* or self inflict* or selfinjur* or self injur* or selfmutilat* or self mutilat* or selfpoison* or self poison* or suicide*).tw
18. exp school suspension/
19. exp school expulsion/
20. exp Educational Attainment Level/
21. (manage* adj move*).tw
22. (terminat* adj5 school*).tw
23. (exclusion or exclude*).tw
24. 'failure to complete'.tw
25. expulsion.tw
26. suspension.tw
27. expel*.tw
28. ((school* or kindergarten or nursery or education) adj4 (attend* or non-attend* or refus* or absen* or school phobi* or truan*).tw
29. 1 or 2 or 3 or 4 or 5 or 6 or 7 or 8 or 9 or 10 or 11
30. 12 or 13 or 14 or 15 or 16 or 17
31. 18 or 19 or 20 or 21 or 22 or 23 or 24 or 25 or 26 or 27 or 28
32. 29 and 30 and 31

For peer review only

EMBASE

1. Child*.tw
2. Exp Child/
3. Adolescen*.tw
4. Exp Adolescent/
5. Student*.tw
6. Exp Students/
7. Youth*.tw
8. Pupil*.tw
9. Schoolchild*.tw
10. (young adj (people or person)).tw
11. teen*.ti,ab.
12. p?diatric*.ti,ab.
13. exp suicide/
14. exp Suicide, Attempted/
15. exp Suicidal ideation/
16. exp suicide attempt/
17. exp suicidal behavior/
18. exp Drug overdose/
19. exp Self-injurious behaviour or exp Self Mutilation/
20. (autoaggress$ or auto aggress$ or automutilat$ or auto mutilat$ or cutt$ or overdose$ or (self adj2 cut$) or selfdestruct$ or self destruct$ or selfharm$ or self harm$ or selfimmolat$ or self immolat$ or selfinflict$ or self inflict$ or selfinjur$ or self injur$ or selfmutilat$ or self mutilat$ or selfpoison$ or self poison$ or suicid$).ti,ab.
21. (expulsion adj5 school*).ti,ab
22. (Suspen* adj5 school*).ti,ab.
23. (manage* adj move*).ti,ab.
24. (Terminat* adj5 school*).ti,ab.
25. (expel* adj5 school*).ti,ab.
26. ((exclusion or exclude*) adj6 school*).ti,ab.
27. ("failure to complete" adj5 (school* or educat*)).ti,ab.
28. ((school* or kindergarten or nursery or education*) adj4 (attend* or non-attend* or refus* or absen* or school phobi* or truan*)).tw.
29. exp school attendance/
30. 1 or 2 or 3 or 4 or 5 or 6 or 7 or 8 or 9 or 10 or 11 or 12
31. 13 or 14 or 15 or 16 or 17 or 18 or 19 or 20
32. 21 or 22 or 23 or 24 or 25 or 26 or 27 or 28 or 29
33. 30 and 31 and 32

For peer review only

Medline

1. child*.tw.
2. exp Child/
3. adolescen*.tw.
4. exp Adolescent/
5. student*.tw.
6. exp Students/
7. youth*.tw.
8. pupil*.tw.
9. schoolchild*.tw.
10. (young adj (people or person)).tw.
11. teen*.ti,ab.
12. p?diatric*.ti,ab.
13. exp suicide/
14. exp Suicide, Attempted/
15. exp Suicidal Ideation/
16. exp Drug Overdose/
17. exp Self-Injurious Behavior/ or exp Self Mutilation/
18. (autoaggress$ or auto aggress$ or automutilat$ or auto mutilat$ or cutt$ or overdose$ or (self adj2 cut$) or selfdestruct$ or self destruct$ or selfharm$ or self harm$ or selfimmolat$ or self immolat$ or selfinflict$ or self inflict$ or selfinjur$ or self injur$ or selfmutilat$ or self mutilat$ or selfpoison$ or self poison$ or suicid$).ti,ab.
19. (expulsion adj5 school*).ti,ab.
20. (Suspen* adj5 school*).ti,ab.
21. (manage* adj move*).ti,ab.
22. Terminat* adj5 school*).ti,ab.
23. (expel* adj5 school*).ti,ab.
24. ((exclusion or exclude*) adj6 school*).ti,ab.
25. ("failure to complete" adj5 (school* or educat*)).ti,ab.
26. ((school* or kindergarten or nursery or education*) adj4 (attend* or non-attend* or refus* or absen* or school phobi* or truan*)).tw.
27. 1 or 2 or 3 or 4 or 5 or 6 or 7 or 8 or 9 or 10 or 11 or 12
28. 13 or 14 or 15 or 16 or 17 or 18
29. 19 or 20 or 21 or 22 or 23 or 24 or 25 or 26
30. 27 and 28 and 29

For peer review only

ERIC

S47	S18 AND S43 AND S46
S46	S19 OR S20 OR S21 OR S22 OR S44 OR S45
S45	AB (autoaggress* OR “auto aggress*” OR automutilat* OR “auto mutilate*” OR cutt* OR overdos* OR (self N2 cut*) OR self-destruct* OR “self destruct*” OR selfharm* OR “self harm*” OR selfimmolat* OR “self immolate*” OR selfinflict* OR “self inflict*” OR selfinjur* OR “self injur*” OR selfmutilat* OR “self mutilate*” OR selfpoison* OR “self poison*” OR suicid*)
S44	AB (autoaggress* OR “auto aggress*” OR automutilat* OR “auto mutilate*” OR cutt* OR overdos* OR (self N2 cut*) OR self-destruct* OR “self destruct*” OR selfharm* OR “self harm*” OR selfimmolat* OR “self immolate*” OR selfinflict* OR “self inflict*” OR selfinjur* OR “self injur*” OR selfmutilat* OR “self mutilate*” OR selfpoison* OR “self poison*” OR suicid*)
S43	S23 OR S24 OR S25 OR S26 OR S27 OR S28 OR S29 OR S30 OR S31 OR S32 OR S33 OR S34 OR S35 OR S36 OR S37 OR S38 OR S39 OR S40 OR S41 OR S42
S42	DE "Withdrawal (Education)"
S41	DE "Attendance" OR DE "Average Daily Attendance" OR DE "College Attendance"
S40	DE "Suspension"
S39	DE "Expulsion"
S38	AB expel*
S37	TI expel*
S36	AB suspension
S35	TI suspension
S34	AB expulsion
S33	TI expulsion
S32	AB "failure to complete"
S31	TI "failure to complete"
S30	AB (exclusion OR exclude*)
S29	TI (exclusion OR exclude*)
S28	AB (terminat* N5 school*)
S27	TI (terminat* N5 school*)
S26	AB (manage* N1 move*)
S25	TI (manage* N1 move*)
S24	AB ((school* OR kindergarten OR nursery OR education*) N4 (attend* OR non-attend* OR refus* OR absen* OR school phobi* OR truan* OR dropout*))
S23	TI ((school* OR kindergarten OR nursery OR education*) N4 (attend* OR non-attend* OR refus* OR

For peer review only

	absen* OR school phobi* OR truan* OR dropout*))
S22	DE "Self Destructive Behavior"
S21	AB suicid*
S20	TI suicid*
S19	DE "Suicide"
S18	S1 OR S2 OR S3 OR S4 OR S5 OR S6 OR S7 OR S8 OR S9 OR S10 OR S11 OR S12 OR S13 OR S14 OR S15 OR S16 OR S17
S17	AB (young N1 (people OR person))
S16	TI (young N1 (people OR person))
S15	AB schoolchild*
S14	TI schoolchild*
S13	AB pupil*
S12	TI pupil*
S11	AB youth*
S10	TI youth*
S9	DE "Students"
S8	AB student*
S7	TI student*
S6	DE "Adolescents"
S5	AB adolescen*
S4	TI adolescen*
S3	AB child*
S2	TI child*
S1	DE "Child Health" OR DE "Children"

BEI

S49	S20 AND S25 AND S48
S48	S26 OR S27 OR S28 OR S29 OR S30 OR S31 OR S32 OR S33 OR S34 OR S35 OR S36 OR S37 OR S38 OR S39 OR S40 OR S41 OR S42 OR S43 OR S44 OR S45 OR S46 OR S47
S47	DE "SCHOOL dropouts"
S46	DE "COLLEGE attendance"
S45	DE "SCHOOL attendance"
S44	DE "STUDENT suspension"
S43	DE "EXCLUSION from school"
S42	DE "STUDENT expulsion"
S41	AB expel*

For peer review only

S40	TI expel*
S39	AB suspension
S38	TI suspension
S37	AB expulsion
S36	TI expulsion
S35	AB "failure to complete"
S34	TI "failure to complete"
S33	AB (exclusion OR exclude*)
S32	TI (exclusion OR exclude*)
S31	AB (terminat* N5 school*)
S30	TI (terminat* N5 school*)
S29	AB (manage* N1 move*)
S28	TI (manage* N1 move*)
S27	AB ((school* OR kindergarten OR nursery OR education*) N4 (attend* OR non-attend* OR refus* OR absen* OR school phobi* OR truan* OR dropout*))
S26	TI ((school* OR kindergarten OR nursery OR education*) N4 (attend* OR non-attend* OR refus* OR absen* OR school phobi* OR truan* OR dropout*))
S25	S21 OR S22 OR S23 OR S24
S24	AB (autoaggress* OR “auto aggress*” OR automutilat* OR “auto mutilate*” OR cutt* OR overdos* OR (self N2 cut*) OR self-destruct* OR “self destruct*” OR selfharm* OR “self harm*” OR selfimmolat* OR “self immolate*” OR selfinflict* OR “self inflict*” OR selfinjur* OR “self injur*” OR selfmutilat* OR “self mutilate*” OR selfpoison* OR “self poison*” OR suicid*)
S23	AB (autoaggress* OR “auto aggress*” OR automutilat* OR “auto mutilate*” OR cutt* OR overdos* OR (self N2 cut*) OR self-destruct* OR “self destruct*” OR selfharm* OR “self harm*” OR selfimmolat* OR “self immolate*” OR selfinflict* OR “self inflict*” OR selfinjur* OR “self injur*” OR selfmutilat* OR “self mutilate*” OR selfpoison* OR “self poison*” OR suicid*)
S22	AB suicid*
S21	TI suicid*
S20	S1 OR S2 OR S3 OR S4 OR S5 OR S6 OR S7 OR S8 OR S9 OR S10 OR S11 OR S12 OR S13 OR S14 OR S15 OR S16 OR S17 OR S18 OR S19
S19	AB (young N1 (people OR person))
S18	TI (young N1 (people OR person))
S17	AB schoolchild*
S16	TI schoolchild*
S15	AB pupil*
S14	TI pupil*

For peer review only

S13	AB youth*
S12	TI youth*
S11	DE "STUDENTS"
S10	AB student*
S9	TI student*
S8	DE "ADOLESCENT psychology"
S7	(DE "TEENAGERS") OR (DE "ADOLESCENCE")
S6	AB adolescen*
S5	TI adolescen*
S4	AB child*
S3	TI child*
S2	(DE "CHILD psychology") OR (DE "CHILD psychiatry")
S1	(DE "CHILDREN") OR (DE "CHILDREN -- Health")

For peer review only

Data Extraction

Reference:

Self-harm variable
Type and duration:

Binary/other:

Method of ascertainment:

Attendance/exclusion variable
Type and duration:

Binary/other:

Method of ascertainment:

Covariates:

N subjects (or cases/controls):

Characteristics of cohort:

Country:

Age (mean/median and range):

Study design:

Time period of data collection:

Odds ratio(s) or other effect estimate(s):

Confidence interval(s):

P Value(s):

Notes about results:

For peer review only

For quality assessment (See risk of bias tools for more information)

<u>Cross sectional:</u>
Representativeness of sample (0-1):
Sample size (0-1):
Non-respondents (0-1):
Ascertainment of attendance/exclusion variable (0-1):
Comparability (0-2):
Ascertainment of self-harm variable (0-1):
Statistical reporting (0-1):
Clarity of variable definitions (0-2):

<u>Case control:</u>
Case definition (0-1):
Representativeness of cases (0-1):
Selection of controls (0-1):
Definition of controls (0-1):
Comparability (0-2):
Ascertainment of attendance/exclusion variable (0-1):
Same ascertainment method for cases and controls (0-1):
Non-response rate (0-1):
Sample size (0-1):
Statistical reporting (0-1):
Clarity of variable definitions (0-2):

<u>Cohort:</u>
Representativeness of exposed cohort (0-1):
Selection of non-exposed cohort (0-1):
Definition of controls (0-1):
Ascertainment of attendance/exclusion variable (0-1):
Outcome not present at start (0-1):
Comparability (0-2):
Ascertainment of self-harm variable (0-1):
Follow-up long enough (0-1):
Adequacy of follow-up (0-1):
Sample size (0-1):
Statistical reporting (0-1):
Clarity of variable definitions (0-2):

For peer review only

Adapted Newcastle-Ottawa Quality Assessment Scales

Cohort Studies

Note: A study can be awarded a maximum of one point for each numbered item with the Selection and Exposure categories. A maximum of two points can be given for Comparability.

Selection (maximum 4)

1. Representativeness of the cohort
   a. Truly representative of the average in the target population (random sample or whole population)*
   b. Somewhat representative of the average in the target population (purposive sampling of representative schools or evidence that the sample is representative of the source population)*
   c. Selected group of users
   d. No description of the derivation of the cohort
2. Selection of the non-exposed cohort
   a. Drawn from the same community as the exposed cohort *
   b. Drawn from a different source
   c. No description of the derivation of the non-exposed cohort
3. Ascertainment of exposure (absence or exclusion)
   a. School administrative records*
   b. Reported by school staff*
   c. Other collateral or self-report
   d. No description
4. Demonstration that outcome of interest was accounted for or not present at start of study
   a. Yes *
   b. No

Comparability (maximum 2)

1. Comparability of cohorts on the basis of the design or analysis
   a. Study controls for age and gender (or analysis separated by gender) *
   b. Study controls for any additional factor *

Outcome (maximum 3)

1. Ascertainment of outcome (self-harm or suicidality)
   a. Structured interview or written self-report*
   b. Clinical record*
   c. Reported by school or collateral
   d. No description
2. Was follow-up long enough for outcomes to occur?
   a. Yes (>1 year) *
   b. No
3. Adequacy of follow-up of cohorts
   a. Complete follow-up – all subjects accounted for *
   b. Subjects lost to follow-up unlikely to introduce bias – small number lost (<20%) or attrition described and accounted for in analysis *
   c. Follow up rate not adequate and no description of those lost

For peer review only

d. No statement

Note: A study can be awarded a maximum of one point for each numbered item for sample size and statistical test. A maximum of two points can be given for clear variables.

Additional parameters: Not in NOS, taken from cross sectional NOS:

1. Sample size (Maximum 1)
   a. Justified and satisfactory *
   b. Adequately powered to detect a difference (at least 10 events per variable in multivariate analyses)*
   c. Not justified

2. Statistical test (Maximum 1)
   a. The statistical test used to analyse the data is clearly described and appropriate, and the measurement of the association is presented as either an OR, CI and P value or a beta coefficient, SE and P value*
   b. The statistical test is not appropriate, not described or incomplete

Further additional parameter:

1. Clear variables (Maximum 2)
   a. Both variables clearly defined including time period for measurement (2)
   b. One or other variable clearly defined including time period for measurement (1)
   c. Neither variable clearly defined including time period for measurement (0)

Case-Control Studies

Note: A study can be awarded a maximum of one point for each numbered item with the Selection and Exposure categories. A maximum of two points can be given for Comparability.

Selection (maximum 4)

5. Is the case definition adequate? (self-harm or suicidality)
   a. Structured interview or written self-report*
   b. Clinical record*
   c. Reported by school or collateral
   d. No description
6. Representativeness of the cases
   a. Consecutive or obviously representative series of cases *
   b. Potential for selection biases or not stated
7. Selection of controls
   a. Community controls from source population*
   b. Clinical controls if clinical source population*
   c. Not extracted from same source population
   d. No description
8. Definition of controls

For peer review only

   a. No history of outcome (NB if cases have new (not necessarily first) occurrence of outcome, controls with previous occurrences should not be excluded) *
   b. No description of source

Comparability (maximum 2)

2. Comparability of cases and controls on the basis of the design or analysis
   a. Study controls for age and gender (or analysis separated by gender)*
   b. Study controls for any additional factor *

Exposure (maximum 3)

4. Ascertainment of exposure (absence or exclusion)
   a. School administrative records*
   b. Reported by school staff*
   c. Other collateral or self-report
   d. No description
5. Same method of ascertainment for cases and controls
   a. Yes *
   b. No
6. Non-response rate
   a. Same rate for both groups *
   b. Non-response or missing values should be <20% and accounted for in analysis*
   c. Non respondents described
   d. Rate different and no designation

Note: A study can be awarded a maximum of one point for each numbered item for sample size and statistical test. A maximum of two points can be given for clear variables.

Additional parameters: Not in NOS, taken from cross sectional NOS:

3. Sample size (Maximum 1)
   a. Justified and satisfactory *
   b. Adequately powered to detect a difference (at least 10 events per variable in multivariate analyses)*
   c. Not justified

4. Statistical test (Maximum 1)
   a. The statistical test used to analyse the data is clearly described and appropriate, and the measurement of the association is presented as either an OR, CI and P value or a beta coefficient, SE and P value*
   b. The statistical test is not appropriate, not described or incomplete

Further additional parameter:

2. Clear variables (Maximum 2)
   a. Both variables clearly defined including time period for measurement (2)
   b. One or other variable clearly defined including time period for measurement (1)
   c. Neither variable clearly defined including time period for measurement (0)

For peer review only

Cross-Sectional Studies

Note: A study can be awarded a maximum of one point for each numbered item with the Selection and Exposure categories. A maximum of two points can be given for Comparability.

Selection (maximum 4)

9. Representativeness of the sample
    a. Truly representative of the average in the target population (random sample or whole population) *
    b. Somewhat representative of the average in the target population (purposive sampling of representative schools or evidence that the sample is representative of the source population) *
    c. Selected group of users
    d. No description of the sampling strategy
10. Sample size
    a. Justified and satisfactory *
    b. Adequately powered to detect a difference (at least 10 events per variable in multivariate analyses)*
    c. Not justified
11. Non-respondents
    a. Comparability between respondents and non-respondents characteristics is established, and the response rate is satisfactory (>60%)*
    b. The response rate is unsatisfactory, or the comparability between respondents and non-respondents is unsatisfactory
    c. No description of the response rate or the characteristics of the responders and non-responders
12. Ascertainment of the exposure (absence or exclusion)
    a. School administrative records*
    b. Reported by school staff*
    c. Other collateral or self-report
    d. No description

Comparability (maximum 2)

3. The subjects in different outcome groups are comparable, based on the study design or analysis. Confounding factors are controlled
    a. Study controls for age and gender (or analysis separated by gender)*
    b. Study controls for any additional factor *

Outcome (maximum 2)

7. Assessment of the outcome (self-harm or suicidality)
    a. Structured interview or written self-report*
    b. Clinical record*
    c. Reported by school or collateral
    d. No description

8. Statistical test

For peer review only

a. The statistical test used to analyse the data is clearly described and appropriate, and the measurement of the association is presented as either an OR, CI and P value or a beta coefficient, SE and P value*
b. The statistical test is not appropriate, not described or incomplete

---

Additional parameter not in NOS:

3. Clear variables (Maximum 2)
    a. Both variables clearly defined including time period for measurement (2)
    b. One or other variable clearly defined including time period for measurement (1)
    c. Neither variable clearly defined including time period for measurement (0)

For peer review only

Reporting checklist for protocol of a systematic review

		Reporting Item	Page Number
Identification	#1a	Identify the report as a protocol of a systematic review	1
Update	#1b	If the protocol is for an update of a previous systematic review, identify as such	n/a
	#2	If registered, provide the name of the registry (such as PROSPERO) and registration number	1
Contact	#3a	Provide name, institutional affiliation, e-mail address of all protocol authors; provide physical mailing address of corresponding author	1 & 7-8
Contribution	#3b	Describe contributions of protocol authors and identify the guarantor of the review	7
	#4	If the protocol represents an amendment of a previously completed or published protocol, identify as such and list changes; otherwise, state plan for documenting important protocol amendments	n/a
Sources	#5a	Indicate sources of financial or other support for the review	7
Sponsor	#5b	Provide name for the review funder and / or sponsor	n/a
Role of sponsor or funder	#5c	Describe roles of funder(s), sponsor(s), and / or institution(s), if any, in developing the protocol	n/a
Rationale	#6	Describe the rationale for the review in the context of what is already known	2-3
Objectives	#7	Provide an explicit statement of the question(s) the review will address with reference to participants, interventions, comparators, and outcomes (PICO)	3
Eligibility criteria	#8	Specify the study characteristics (such as PICO, study design, setting, time frame) and report characteristics	3-4

For peer review only

		(such as years considered, language, publication status) to be used as criteria for eligibility for the review	
Information sources	#9	Describe all intended information sources (such as electronic databases, contact with study authors, trial registers or other grey literature sources) with planned dates of coverage	4
Search strategy	#10	Present draft of search strategy to be used for at least one electronic database, including planned limits, such that it could be repeated	Supplementary File 1
Study records - data management	#11a	Describe the mechanism(s) that will be used to manage records and data throughout the review	5
Study records - selection process	#11b	State the process that will be used for selecting studies (such as two independent reviewers) through each phase of the review (that is, screening, eligibility and inclusion in meta-analysis)	5
Study records - data collection process	#11c	Describe planned method of extracting data from reports (such as piloting forms, done independently, in duplicate), any processes for obtaining and confirming data from investigators	5
Data items	#12	List and define all variables for which data will be sought (such as PICO items, funding sources), any pre-planned data assumptions and simplifications	5 & Supplementary File 2
Outcomes and prioritization	#13	List and define all outcomes for which data will be sought, including prioritization of main and additional outcomes, with rationale	3-5
Risk of bias in individual studies	#14	Describe anticipated methods for assessing risk of bias of individual studies, including whether this will be done at the outcome or study level, or both; state how this information will be used in data synthesis	5 & Supplementary File 3
Data synthesis	#15a	Describe criteria under which study data will be quantitatively synthesised	6
	#15b	If data are appropriate for quantitative synthesis, describe planned summary measures, methods of	6

For peer review only

		handling data and methods of combining data from studies, including any planned exploration of consistency (such as I2, Kendall's τ)	
	#15c	Describe any proposed additional analyses (such as sensitivity or subgroup analyses, meta-regression)	6
	#15d	If quantitative synthesis is not appropriate, describe the type of summary planned	6
Meta-bias(es)	#16	Specify any planned assessment of meta-bias(es) (such as publication bias across studies, selective reporting within studies)	6
Confidence in cumulative evidence	#17	Describe how the strength of the body of evidence will be assessed (such as GRADE)	6

The PRISMA-P checklist is distributed under the terms of the Creative Commons Attribution License CC-BY 4.0. This checklist was completed on 27. April 2018 using http://www.goodreports.org/, a tool made by the EQUATOR Network in collaboration with Penelope.ai

For peer review only

BMJ Open

Poor school attendance and exclusion: a systematic review protocol on educational risk factors for self-harm and suicidal behaviours

Journal:	*BMJ Open*
Manuscript ID	bmjopen-2018-023953.R1
Article Type:	Protocol
Date Submitted by the Author:	17-Sep-2018
Complete List of Authors:	Epstein, Sophie; South London and Maudsley NHS Foundation Trust, NIHR Maudsley Biomedical Research Centre; King's College London, Department of Child and Adolescent Psychiatry<br>Roberts , Emmert; King's College London, National Addiction Centre & Department of Psychological Medicine; South London and Maudsley NHS Foundation Trust<br>Sedgwick, Rosemary; South London and Maudsley NHS Foundation Trust; King's College London, Department of Child and Adolescent Psychiatry<br>Finning, Katie; University of Exeter Medical School<br>Ford, Tamsin; University of Exeter Medical School<br>Dutta, Rina; Kings College London, Department of Psychological Medicine ; South London and Maudsley NHS Foundation Trust, NIHR Maudsley Biomedical Research Centre<br>Downs, Johnny; Kings College London, Department of Child and Adolescent Psychiatry ; South London and Maudsley NHS Foundation Trust, NIHR Maudsley Biomedical Research Centre
**Primary Subject Heading**:	Mental health
Secondary Subject Heading:	Public health
Keywords:	Child & adolescent psychiatry < PSYCHIATRY, Suicide & self-harm < PSYCHIATRY, School mental health, Systematic review protocol

SCHOLARONE™
Manuscripts

Poor school attendance and exclusion: a systematic review protocol on educational risk factors for self-harm and suicidal behaviours

Sophie Epstein[1,2,3]*, Emmert Roberts[2,4,5], Rosemary Sedgwick[2,3], Katie Finning[6], Tamsin Ford[6], Rina Dutta[1,2,5], Johnny Downs[1,2,3]

1. NIHR Maudsley Biomedical Research Centre, South London and Maudsley NHS Foundation Trust
2. South London and Maudsley NHS Foundation Trust
3. Department of Child and Adolescent Psychiatry, King's College London
4. National Addiction Centre, King's College London
5. Department of Psychological Medicine, King's College London
6. University of Exeter Medical School

*Corresponding author

Abstract

Introduction

Schools have an important role in recognising and preventing self-harm and suicidal behaviour in their students, however little is known about which educational factors are associated with heightened risk. We will systematically review the existing evidence on two key educational performance indicators that are routinely collected by school administrative systems: school attendance and exclusion. We will investigate their association with self-harm and suicidal behaviour in school-age children and adolescents. Knowledge of this association could help to inform suicide prevention strategies at clinical, school and population level.

Methods and analysis

We will conduct a systematic search of Medline, EMBASE, PsycINFO, British Education Index and Education Resources Information Centre (ERIC) from 01/01/1990, and conduct a manual search for additional references. We aim to identify studies that explore the association between poor school attendance or exclusion and self-harm or suicidal behaviours in school-age children and adolescents. Two independent reviewers will screen titles, abstracts and full text documents and independently extract relevant data for analysis. Study quality will be assessed using a modified Newcastle-Ottawa Scale. A descriptive analysis will be performed, and where appropriate, results will be combined in meta-analyses.

Ethics and dissemination

This is a systematic review of published literature, and therefore ethical approval will not be sought. We will publish reports in health and education journals, present our work at conferences focused on school mental health and communicate our findings to practitioners and managers in public health, education and child mental health.

Registration details

The review is registered on PROSPERO. ID CRD42018088608

For peer review only

Strengths and limitations of this study

1. This will be the first systematic review to investigate poor school attendance and exclusion as risk factors for self-harm and suicidal behaviour in school-age children and adolescents.

2. The methods involve a systematic search of databases and other sources from both health and education fields, including comprehensive forward and backward citation searching and contacting key authors in the field.

3. The wide inclusion criteria mean that this review will serve as a broad overview of the questions of interest and will allow identification of particular gaps in the existing evidence base.

4. The use of wide inclusion criteria may also create challenges in synthesising the evidence as constructs described in each study may not be comparable.

5. This systematic review does not include non-peer reviewed literature and only includes studies published in the English language and therefore may not report an entirely comprehensive view of the literature on this topic.

Introduction

Self-harm and suicidal behaviour in children and adolescents are important public health issues. International community-based studies have reported a prevalence of self-harm in adolescents of approximately 10%.[1] Suicide is the second most common cause of death worldwide for the 10-24 age group [2] and national studies in several countries have found that on average between 1 and 10% of adolescents report having attempted suicide during their lifetime.[3-7] Self-harm and attempted suicide are amongst the strongest predictors of completed suicide [8 9] and are strongly associated with mental disorders including depression and anxiety.[6]

In the UK, the focus of current child and adolescent mental health policy is on developing closer collaborations between the health and education sectors and supporting schools to develop capacity to play a greater role in supporting and promoting pupils' mental health. [10] This includes a role in early identification of those at risk of mental health difficulties or risky behaviours including self-harm. Currently, many school-based suicide prevention programmes exist, but most constitute universal prevention rather than using strategies that target particular at-risk groups.[11] In the UK, it is unclear how broadly such interventions are implemented or how at-risk groups are targeted (Evans, Parker, Russell et al, Adolescent self-harm prevention and intervention in secondary schools: A survey of staff in England and Wales, In Submission).

Any policy development needs to be informed by robust evidence on the factors that could help to identify those children and adolescents at greater risk of self-harm or suicidal behaviours. A number of risk factors for self-harm and suicidal behaviour in children and adolescents have been identified, which include both psychological factors such as mental disorders, drug and alcohol misuse; personality characteristics such as impulsivity; as well as socio-demographic factors such as female sex for self-harm, and male sex for completed suicide, low socio-economic status, adverse childhood experiences, family discord and bullying.[1 12 13] Risk profiles are also found to vary between subgroups when stratified by certain socio-demographic factors such as gender, age and ethnicity.[1 3 14]

A small number of studies have reported an association between suicidal phenomena and poor school attendance, actual and perceived educational attainment, poor school

For peer review only - http://bmjopen.bmj.com/site/about/guidelines.xhtml

connectedness and experiences, and negative attitudes towards school.[1 12 15-20] There is also evidence of higher rates of suicide attempts among young adults with a history of poor school performance [21 22] but a lower risk amongst those who attended schools with a lower average academic performance.[22]

Beyond this however, educational factors, which may be particularly pertinent in this age-group, are yet to be explored in any detail. This is surprising, given that data on these factors are routinely collected by education systems in many high income countries.

Although many systematic reviews that explore risk factors for self-harm and suicidal behaviour among children and adolescents have been published, we have not been able to identify any which specifically report on educational risk factors, and none which attempt to comprehensively identify the literature that explores poor school attendance and school exclusion as possible risk factors for self-harm and suicidal behaviour.

In this systematic review, poor school attendance and exclusion from school are hypothesised to be two potentially important risk factors for self-harm and suicidal behaviours in school-age populations. We seek to answer the question; are poor attendance or exclusion from school associated with self-harm and/or suicidal behaviour in school-age children and adolescents? We also aim to explore whether this association persists after accounting for other potentially confounding socio-demographic risk factors for self-harm and suicidal behaviour such as age, gender and ethnicity.

Methods

This protocol follows the Preferred Reporting Items for Systematic review and Meta-Analysis Protocols (PRISMA-P)[23] guidelines (Supplementary File 1 – PRISMA-P Checklist) and is registered on PROSPERO, an international register of systematic reviews ID=CRD42018088608.[24] Any changes to the protocol will be recorded on PROSPERO.

Eligibility criteria:

Population

Eligible participants will be those aged 4-18 and attending school at the point of enrolment into the study or where poor attendance or exclusion from school and at least a proportion of the self-harm or suicidal behaviour are measured during this age range. The reason for this choice of age range is that it constitutes the school age population in most countries.

Types of studies

Any observational studies (cohort, longitudinal, case control, cross sectional) published from 1990 onwards will be included. Qualitative studies, case reports, intervention studies and comments/editorials will be excluded. Studies conducted in any country will be included, provided they are published in English. The reason for exclusion of older studies, is that due to the changing nature of educational systems and social contexts within which self-harm occurs, such studies are less relevant to the present day. Only peer reviewed papers will be included, and only those where the full text of the study is available. Grey literature will not be included.

Exposure and outcome variables

Any study that investigates a possible association between poor school attendance or school exclusion and self-harm or suicidal behaviour will be included. Studies will only be

For peer review only - http://bmjopen.bmj.com/site/about/guidelines.xhtml

included if there is a comparison group. In order to be included, a study will need to report poor school attendance or exclusion as an exposure and self-harm or suicidal behaviour as an outcome. Exceptions to this rule are cross sectional studies that consider self-harm or suicidal behaviour as an exposure and poor attendance or exclusion as an outcome, and which report results for univariate analyses.

The existing literature on self-harm and suicidal behaviour is extremely heterogeneous in terms of the terminology used. We will therefore consider a wide definition to encompass this variation, which will include any form of self-harm or thoughts about self-harm, and any form of suicidal behaviour including suicidal thoughts or ideation, plans, attempts, or completed suicide. The data on this variable can be ascertained by any method including written self-reports, interviews, collateral reports or clinical or other secure records.

In terms of poor school attendance and exclusion, again a broad definition will be considered as terminology used in the international literature varies widely. Acceptable variables will include any form of exclusion or suspension from school, temporary or permanent, over any period of time. Any authorised or unauthorised non-attendance at school (amongst pupils enrolled in school) including school refusal, school phobia, truancy or long term absence due to ill health will be included. Studies using 'school dropout' as an exposure of interest will be excluded as this considered a different construct to poor school attendance. Additionally, those studies that consider attendance in relation to whether the child is or is not enrolled at school, will be excluded. Attendance in relation to being on school roll or not taps a different set of issues and is of greatest relevance to populations in low and middle income countries where some children do not attend school for financial reasons or due to lack of education provision. Any method of data collection will be acceptable, including self or collateral reports or secure records.

Search methods

The following health and education databases will be searched as follows:

- Medline (OvidSP platform)
- Embase (OvidSP platform)
- PsycINFO (OvidSP platform)
- British Education Index (EBSCO platform)
- Education Resources Information Centre (EBSCO platform)

Databases will be searched from 01/01/1990 until present. The search strategy is described below.

Citation lists of included studies will then be checked for other relevant studies (backward citation searching), and forward citation searching will be conducted by systematically looking through lists of papers which have subsequently cited the included studies. Where possible, Web of Science will be used for this purpose, and in situations where the paper is not listed on Web of Science, Google Scholar will be used. We will also look through the reference lists of existing systematic reviews on similar topics to identify any further relevant papers. If any key journals are identified during the initial searches, we will hand search these journals to look for additional relevant papers.

Once a final list of papers has been generated using the above methods, experts in the field will be contacted to identify any additional papers.

For peer review only

Search strategy for electronic databases

The search strategies have been developed to include both keywords and thesaurus terms relevant to each respective database. For keyword searching, truncation and wildcards are used as necessary to allow for linguistic variations. Full search strategies can be found in Supplementary File 2. The included terms refer to the following concepts:

- Population (e.g. child, adolescent)
- Attendance/exclusion (e.g. absence, truancy, exclusion)
- Self-harm/suicidal behaviour (e.g. suicidal, self-harm)

Due to the wide range of possible terms to describe self-harm, the list of terms used for this part of the search strategy has been taken from a recent review on suicide and self-harm published in the British Journal of Psychiatry.[25] Similarly, the terms for poor school attendance were taken from a related systematic review protocol which was developed in consultation with an information specialist as well as experts in the fields of child mental health and education.[26]

Selection of references

Titles and abstracts generated from the searches of electronic databases will be exported to Endnote X8 software and duplicates removed. The references will then be screened for eligibility by two independent reviewers. Any uncertainties will be discussed between the two reviewers (SE and RS). Any discrepancies between the decisions of the two reviewers will be initially discussed between them, and if a consensus cannot be reached, a third reviewer (JD) will adjudicate. Included full texts will be obtained and screened by two reviewers and again any disagreement will be discussed with a third reviewer.

Data extraction

Data will be extracted by two independent reviewers (SE and ER) according to an agreed data extraction form (Supplementary File 3). Data to be extracted will include: Definitions of exposure and outcome variables and methods of ascertainment, covariates included in analysis, characteristics of the cohort including age and gender balance of participants, country, study design, relevant results (including effect estimates, confidence intervals, and p-values, where reported) and information for the assessment of study quality/risk of bias. Where two studies report data from the same cohort, the larger sample, or if the same sample, the study with the longest follow up will be used to ensure participants are not double counted.

Quality assessment

Risk of bias within the included studies will be assessed using modified Newcastle-Ottawa Scales (NOS) for assessment of risk of bias in observational studies.[27] The modified scales can be found in Supplementary File 4. This will be carried out by two independent reviewers (SE and ER). As there is no original version of the NOS for cross-sectional studies, an adapted version used in previously published literature, will be used.[28] Some items on the NOS scales have been adapted for relevance to this research question, in particular the items relating to acceptable methods of ascertainment of the exposure and outcome variables. Further key quality parameters will be assessed in addition to the NOS items as follows: appropriate sample size, appropriate statistical tests and clarity of reporting of exposure and outcome variables. Any discrepancies will be discussed between the two reviewers, and if a consensus cannot be reached, a third reviewer will adjudicate.

For peer review only - http://bmjopen.bmj.com/site/about/guidelines.xhtml

Analysis

Included studies will be described according to their respective characteristics in the text and in tables, and the results of the studies will be discussed in a narrative synthesis, bringing together studies according to particular outcomes, exposures or type of participant where possible. Results will be standardised where possible by reporting or calculating odds ratios. If any two or more studies use sufficiently similar methodology, exposure and outcome variables, meta-analyses will be conducted. A random effects model will be used, due to the likelihood of considerable heterogeneity between studies, and we will present a summary effect with an associated 95% confidence interval and P value. The level of statistical heterogeneity will be determined using the I^2 statistic. Where there is a large enough number of studies within a meta-analysis, publication bias will be assessed using funnel plots and Eggers test. An assessment of the strength of the evidence per outcome will be conducted using the Grading of Recommendations Assessment, Development and Evaluation (GRADE) approach adapted for non-randomised studies. Where possible, results stratified by gender will be presented and if the data within studies allows, we will also report subgroup analyses by age and ethnicity.

Findings from all studies will be reported and included in the descriptive analysis regardless of their risk of bias. The quality of included studies, as assessed by the NOS as described above, will be taken into consideration in the synthesis and analysis of the results, in terms of the strength of the evidence within each group of studies considered. We will conduct a sensitivity analysis removing any papers which score below 60% on the quality assessment scales and report a synthesis of the evidence from the remaining studies to assess whether this has an impact on the overall effect.

Study Status

Data collection was completed in August 2018 and analysis is expected to be completed during October 2018.

Patient and Public Involvement

Consultation with young people, parents and teachers has been used to determine their perception of the importance of educational risk factors for self-harm and suicidal behaviours in this age group. It is based on their perspectives that this systematic review was deemed to be timely and crucial to conduct to inform further research work.

Discussion

This systematic review aims to bring together international evidence on the association between important educational risk factors and a common and important outcome of self-harm and suicidal behaviour in children and adolescents. We are aware that all of these variables constitute broad constructs and the terminology used to describe these constructs varies widely in different contexts. It is therefore likely that there will be challenges involved in synthesising data on this this topic.

An additional challenge is that studies may report poor school attendance and exclusion among many other variables considered as possible risk factors for self-harm and suicidal behaviour. It is possible therefore that terms referring to these particular exposures are not referenced in the titles or abstracts of the studies. We will minimise the risk of missing relevant studies by employing a range of search methods including consultation with experts to identify any additional papers.

For peer review only - http://bmjopen.bmj.com/site/about/guidelines.xhtml

Despite these challenges, this systematic review will be the first to specifically address the question of whether poor school attendance and exclusion are related to self-harm and suicidal behaviour in young people. We will rigorously assess the quality and strength of the evidence, which could generate further research questions, and could ultimately help to inform suicide prevention strategies at clinical, school or population level.

In the UK and many other high income countries, data on school attendance and exclusions are routinely collected for every child in the state education system. Additionally, compared to many other parameters collected, such as socio-economic status and special educational needs, these are relatively objective and standardised measures, which could be used to identify those at higher risk of adverse outcomes, including self-harm and suicidal behaviour. With the opportunity for schools to play an important role in the prevention of self-harm and suicide,[10] it is crucial to know whether these routinely collected factors can be used as predictors of those at higher risk. The presence or absence of an association has important policy implications in terms of the design and implementation of targeted school-based suicide prevention interventions. If evidence on this association (or other important factors which may provide an alternative explanation for this association) is found to be lacking, an urgent need for further research will be highlighted.

Ethics and dissemination

As this is a systematic review of published literature, ethical approval will not be sought. We will publish the protocol and our findings in peer-reviewed journals aimed at school and health commissioners, practitioners and managers. We will present our work at the growing numbers of national and international meetings focused on school mental health. This work will be used to inform our ongoing work with the Department for Education to examine educational risk factor for child and adolescent mental health outcomes, including secondary analyses of the National Pupil Database using linked health and school data.

Author contributions

SE (Guarantor) co-conceived and designed the review with support from other authors, wrote the initial draft of the manuscript, approved the final version and agrees to be accountable for its content.ER contributed to the design of the review, critically revised the manuscript, approved the final version and agrees to be accountable for its content.RS contributed to the design of the review, critically revised the manuscript, approved the final version and agrees to be accountable for its content. KF contributed to the design of the review, critically revised the manuscript, approved the final version and agrees to be accountable for its content.TF contributed to the design of the review, critically revised the manuscript, approved the final version and agrees to be accountable for its content. RD contributed to the design of the review, critically revised the manuscript, approved the final version and agrees to be accountable for its content. JD co-conceived and designed the review with support from other authors, critically revised drafts of the manuscript, approved the final version and agrees to be accountable for its content.

Funding statement

SE has been employed as a National Institute of Health Research (NIHR) Academic Clinical Fellow and currently receives salary support from an MQ Data Science Award and from the Psychiatry Research Trust. ER is funded by a Medical Research Council (MRC) Addiction Research Clinical Training Fellowship. RS is employed as an NIHR Academic Clinical Fellow.

For peer review only - http://bmjopen.bmj.com/site/about/guidelines.xhtml

KF's PhD is funded by the University of Exeter, via the MYRIAD study (Wellcome Trust 107496/Z/15/Z). RD is funded by a Clinician Scientist Fellowship (research project e-HOST-IT) from the Health Foundation in partnership with the Academy of Medical Sciences. JD received support via a MRC Clinical Research Training Fellowship (MR/L017105/1), Psychiatry Research Trust Peggy Pollak Research Fellowship in Developmental Psychiatry and from the NIHR Biomedical Research Centre at South London and Maudsley NHS Foundation Trust and King's College London. This paper represents independent research part funded by the National Institute for Health Research (NIHR) Biomedical Research Centre at South London and Maudsley NHS Foundation Trust and King's College London. The views expressed are those of the authors and not necessarily those of the NHS, the NIHR or the Department of Health and Social Care.

Competing interests declaration
The authors have no competing interests

Correspondence
Dr Sophie Epstein, NIHR Maudsley Biomedical Research Centre Nucleus, PO Box 005, De Crespigny Park, Denmark Hill, London SE5 8AF, United Kingdom.
Email: Sophie.epstein@kcl.ac.uk

Email addresses of other authors
Emmert Roberts: Emmert.roberts@kcl.ac.uk
Rosemary Sedgwick: Rosemary.sedgwick@slam.nhs.uk
Katie Finning: K.Finning@exeter.ac.uk
Tamsin Ford: T.J.Ford@exeter.ac.uk
Rina Dutta: Rina.dutta@kcl.ac.uk
Johnny Downs Johnny.downs@kcl.ac.uk

Supplementary File 1: PRISMA-P checklist

Supplementary File 2: **Search Strategies**

Supplementary File 3: Data extraction form

Supplementary File 4: Quality assessment tools

REFERENCES

1. Hawton K, Saunders KE, O'Connor RC. Self-harm and suicide in adolescents. *Lancet* 2012;379(9834):2373-82. doi: 10.1016/s0140-6736(12)60322-5 [published Online First: 2012/06/26]
2. Patton GC, Coffey C, Sawyer SM, et al. Global patterns of mortality in young people: a systematic analysis of population health data. *Lancet (London, England)* 2009;374(9693):881-92. doi: 10.1016/s0140-6736(09)60741-8 [published Online First: 2009/09/15]
3. Nock MK, Green JG, Hwang I, et al. Prevalence, correlates, and treatment of lifetime suicidal behavior among adolescents: results from the National Comorbidity Survey

For peer review only

Replication Adolescent Supplement. *JAMA Psychiatry* 2013;70(3):300-10. doi: 10.1001/2013.jamapsychiatry.55 [published Online First: 2013/01/11]
4. Hargus E, Hawton K, Rodham K. Distinguishing Between Subgroups of Adolescents Who Self-Harm. *Suicide and Life-Threatening Behavior* 2009;39(5):518-37. doi: 10.1521/suli.2009.39.5.518
5. Madge N, Hewitt A, Hawton K, et al. Deliberate self-harm within an international community sample of young people: comparative findings from the Child & Adolescent Self-harm in Europe (CASE) Study. *Journal of child psychology and psychiatry, and allied disciplines* 2008;49(6):667-77. doi: 10.1111/j.1469-7610.2008.01879.x [published Online First: 2008/03/18]
6. Moran P, Coffey C, Romaniuk H, et al. The natural history of self-harm from adolescence to young adulthood: a population-based cohort study. *The Lancet* 2012;379(9812):236-43. doi: 10.1016/S0140-6736(11)61141-0
7. Kokkevi A, Rotsika V, Arapaki A, et al. Adolescents' self-reported suicide attempts, self-harm thoughts and their correlates across 17 European countries. *Journal of child psychology and psychiatry, and allied disciplines* 2012;53(4):381-9. doi: 10.1111/j.1469-7610.2011.02457.x [published Online First: 2011/09/08]
8. Hawton K, Harriss L. Deliberate self-harm in young people: characteristics and subsequent mortality in a 20-year cohort of patients presenting to hospital. *The Journal of clinical psychiatry* 2007;68(10):1574-83. [published Online First: 2007/10/27]
9. Joiner TE, Jr., Conwell Y, Fitzpatrick KK, et al. Four studies on how past and current suicidality relate even when "everything but the kitchen sink" is covaried. *Journal of abnormal psychology* 2005;114(2):291-303. doi: 10.1037/0021-843x.114.2.291 [published Online First: 2005/05/05]
10. DoH D. Transforming Children and Young People’s Mental Health Provision: a Green Paper. 2017
11. Katz C, Bolton SL, Katz LY, et al. A systematic review of school-based suicide prevention programs. *Depression and anxiety* 2013;30(10):1030-45. doi: 10.1002/da.22114 [published Online First: 2013/05/08]
12. Evans E, Hawton K, Rodham K. Factors associated with suicidal phenomena in adolescents: a systematic review of population-based studies. *Clinical psychology review* 2004;24(8):957-79. doi: 10.1016/j.cpr.2004.04.005 [published Online First: 2004/11/10]
13. Steele M, Doey T. Suicidal Behaviour in Children and Adolescents. Part 1: Etiology and Risk Factors *The Canadian Journal of Psychiatry* 2007;52(6 Supplement 1):21S-33S.
14. Hawton K, Rodham K, Evans E, et al. Deliberate self harm in adolescents: self report survey in schools in England. *BMJ* 2002;325(7374):1207-11. doi: 10.1136/bmj.325.7374.1207
15. Young R, Sweeting H, Ellaway A. Do schools differ in suicide risk? The influence of school and neighbourhood on attempted suicide, suicidal ideation and self-harm among secondary school pupils. *BMC public health* 2011;11:874.
16. Stallard P, Spears M, Montgomery AA, et al. Self-harm in young adolescents (12-16 years): Onset and short-term continuation in a community sample. *BMC Psychiatry* 2013;13:no pagination.
17. Kidger J, Heron J, Leon DA, et al. Self-reported school experience as a predictor of self-harm during adolescence: a prospective cohort study in the South West of England (ALSPAC). *J Affect Disord* 2015;173:163-9. doi: 10.1016/j.jad.2014.11.003 [published Online First: 2014/12/03]

For peer review only - http://bmjopen.bmj.com/site/about/guidelines.xhtml

18. Richardson AS, Bergen HA, Martin G, et al. Perceived academic performance as an indicator of risk of attempted suicide in young adolescents. *Arch Suicide Res* 2005;9(2):163-76. doi: 10.1080/13811110590904016 [published Online First: 2005/07/16]
19. Orozco R, Benjet C, Borges G, et al. Association between attempted suicide and academic performance indicators among middle and high school students in Mexico: results from a national survey. *Child and Adolescent Psychiatry and Mental Health* 2018;12:9. doi: 10.1186/s13034-018-0215-6
20. Lee MT, Wong BP, Chow BW, et al. Predictors of suicide ideation and depression in Hong Kong adolescents: perceptions of academic and family climates. *Suicide Life Threat Behav* 2006;36(1):82-96. [published Online First: 2006/05/09]
21. Kosidou K, Dalman C, Fredlund P, et al. School performance and the risk of suicide attempts in young adults: a longitudinal population-based study. *Psychological medicine* 2014;44(6):1235-43. doi: 10.1017/s0033291713001852 [published Online First: 2013/07/26]
22. Jablonska B, Ostberg V, Hjern A, et al. School effects on risk of non-fatal suicidal behaviour: a national multilevel cohort study. *Soc Psychiatry Psychiatr Epidemiol* 2014;49(4):609-18. doi: 10.1007/s00127-013-0782-z [published Online First: 2013/10/26]
23. Moher D, Shamseer L, Clarke M, et al. Preferred reporting items for systematic review and meta-analysis protocols (PRISMA-P) 2015 statement. *Systematic Reviews* 2015;4(1):1. doi: 10.1186/2046-4053-4-1
24. Booth A, Clarke M, Dooley G, et al. The nuts and bolts of PROSPERO: an international prospective register of systematic reviews. *Systematic Reviews* 2012;1(1):2. doi: 10.1186/2046-4053-1-2
25. Chan MKY, Bhatti H, Meader N, et al. Predicting suicide following self-harm: systematic review of risk factors and risk scales. *The British Journal of Psychiatry* 2016 doi: 10.1192/bjp.bp.115.170050
26. Finning K, Moore D, Ukoumunne OC, et al. The association between child and adolescent emotional disorder and poor attendance at school: a systematic review protocol. *Systematic reviews* 2017;6(1):121. doi: 10.1186/s13643-017-0523-6 [published Online First: 2017/07/01]
27. Wells GA SB, O'Connell D, Peterson J, Welch V, Losos M, Tugwell P. The Newcastle-Ottawa Scale (NOS) for assessing the quality of nonrandomised studies in meta-analyses. *The Ottawa Hospital Research Institute* 2004
28. Herzog R, Alvarez-Pasquin MJ, Diaz C, et al. Are healthcare workers' intentions to vaccinate related to their knowledge, beliefs and attitudes? A systematic review. *BMC public health* 2013;13:154. doi: 10.1186/1471-2458-13-154 [published Online First: 2013/02/21]

For peer review only

Reporting checklist for protocol of a systematic review

		Reporting Item	Page Number
Identification	#1a	Identify the report as a protocol of a systematic review	1
Update	#1b	If the protocol is for an update of a previous systematic review, identify as such	n/a
	#2	If registered, provide the name of the registry (such as PROSPERO) and registration number	1
Contact	#3a	Provide name, institutional affiliation, e-mail address of all protocol authors; provide physical mailing address of corresponding author	1 & 7-8
Contribution	#3b	Describe contributions of protocol authors and identify the guarantor of the review	7
	#4	If the protocol represents an amendment of a previously completed or published protocol, identify as such and list changes; otherwise, state plan for documenting important protocol amendments	n/a
Sources	#5a	Indicate sources of financial or other support for the review	7
Sponsor	#5b	Provide name for the review funder and / or sponsor	n/a
Role of sponsor or funder	#5c	Describe roles of funder(s), sponsor(s), and / or institution(s), if any, in developing the protocol	n/a
Rationale	#6	Describe the rationale for the review in the context of what is already known	2-3
Objectives	#7	Provide an explicit statement of the question(s) the review will address with reference to participants, interventions, comparators, and outcomes (PICO)	3
Eligibility criteria	#8	Specify the study characteristics (such as PICO, study	3-4

For peer review only

		design, setting, time frame) and report characteristics (such as years considered, language, publication status) to be used as criteria for eligibility for the review	
Information sources	#9	Describe all intended information sources (such as electronic databases, contact with study authors, trial registers or other grey literature sources) with planned dates of coverage	4
Search strategy	#10	Present draft of search strategy to be used for at least one electronic database, including planned limits, such that it could be repeated	Supplementary File 1
Study records - data management	#11a	Describe the mechanism(s) that will be used to manage records and data throughout the review	5
Study records - selection process	#11b	State the process that will be used for selecting studies (such as two independent reviewers) through each phase of the review (that is, screening, eligibility and inclusion in meta-analysis)	5
Study records - data collection process	#11c	Describe planned method of extracting data from reports (such as piloting forms, done independently, in duplicate), any processes for obtaining and confirming data from investigators	5
Data items	#12	List and define all variables for which data will be sought (such as PICO items, funding sources), any pre-planned data assumptions and simplifications	5 & Supplementary File 2
Outcomes and prioritization	#13	List and define all outcomes for which data will be sought, including prioritization of main and additional outcomes, with rationale	3-5
Risk of bias in individual studies	#14	Describe anticipated methods for assessing risk of bias of individual studies, including whether this will be done at the outcome or study level, or both; state how this information will be used in data synthesis	5 & Supplementary File 3
Data synthesis	#15a	Describe criteria under which study data will be quantitatively synthesised	6

For peer review only

	#15b	If data are appropriate for quantitative synthesis, describe planned summary measures, methods of handling data and methods of combining data from studies, including any planned exploration of consistency (such as I2, Kendall's τ)	6
	#15c	Describe any proposed additional analyses (such as sensitivity or subgroup analyses, meta-regression)	6
	#15d	If quantitative synthesis is not appropriate, describe the type of summary planned	6
Meta-bias(es)	#16	Specify any planned assessment of meta-bias(es) (such as publication bias across studies, selective reporting within studies)	6
Confidence in cumulative evidence	#17	Describe how the strength of the body of evidence will be assessed (such as GRADE)	6

The PRISMA-P checklist is distributed under the terms of the Creative Commons Attribution License CC-BY 4.0. This checklist was completed on 27. April 2018 using http://www.goodreports.org/, a tool made by the EQUATOR Network in collaboration with Penelope.ai

For peer review only

Search Strategies

PsycINFO

1. Child*.tw
2. Adolescen*.tw
3. Student*.tw
4. Exp Students/
5. Youth*.tw
6. Pupil*.tw
7. Schoolchild*.tw
8. (young adj (people or person).tw
9. teen*.tw
10. p?diatric.tw
11. exp Elementary School Students /
12. exp Suicide/
13. exp Suicide, Attempted/
14. exp Suicidal Ideation/
15. exp Drug Overdose/
16. exp Self-injurious Behaviour or exp Self Mutilation/
17. (autoaggress* or auto aggress* or automutilat* or cutt* or overdose* or (self adj2 cut*) or selfdestruct* or self destruct* or selfharm* or self harm* or selfimmolat* or self immolat* or selfinflict* or self inflict* or selfinjur* or self injur* or selfmutilat* or self mutilat* or selfpoison* or self poison* or suicide*).tw
18. exp school suspension/
19. exp school expulsion/
20. exp Educational Attainment Level/
21. (manage* adj move*).tw
22. (terminat* adj5 school*).tw
23. (exclusion or exclude*).tw
24. ‘failure to complete’.tw
25. expulsion.tw
26. suspension.tw
27. expel*.tw
28. ((school* or kindergarten or nursery or education) adj4 (attend* or non-attend* or refus* or absen* or school phobi* or truan*).tw
29. 1 or 2 or 3 or 4 or 5 or 6 or 7 or 8 or 9 or 10 or 11
30. 12 or 13 or 14 or 15 or 16 or 17
31. 18 or 19 or 20 or 21 or 22 or 23 or 24 or 25 or 26 or 27 or 28
32. 29 and 30 and 31

For peer review only

EMBASE

1. Child*.tw
2. Exp Child/
3. Adolescen*.tw
4. Exp Adolescent/
5. Student*.tw
6. Exp Students/
7. Youth*.tw
8. Pupil*.tw
9. Schoolchild*.tw
10. (young adj (people or person)).tw
11. teen*.ti,ab.
12. p?diatric*.ti,ab.
13. exp suicide/
14. exp Suicide, Attempted/
15. exp Suicidal ideation/
16. exp suicide attempt/
17. exp suicidal behavior/
18. exp Drug overdose/
19. exp Self-injurious behaviour or exp Self Mutilation/
20. (autoaggress$ or auto aggress$ or automutilat$ or auto mutilat$ or cutt$ or overdose$ or (self adj2 cut$) or selfdestruct$ or self destruct$ or selfharm$ or self harm$ or selfimmolat$ or self immolat$ or selfinflict$ or self inflict$ or selfinjur$ or self injur$ or selfmutilat$ or self mutilat$ or selfpoison$ or self poison$ or suicid$).ti,ab.
21. (expulsion adj5 school*).ti,ab
22. (Suspen* adj5 school*).ti,ab.
23. (manage* adj move*).ti,ab.
24. (Terminat* adj5 school*).ti,ab.
25. (expel* adj5 school*).ti,ab.
26. ((exclusion or exclude*) adj6 school*).ti,ab.
27. ("failure to complete" adj5 (school* or educat*)).ti,ab.
28. ((school* or kindergarten or nursery or education*) adj4 (attend* or non-attend* or refus* or absen* or school phobi* or truan*)).tw.
29. exp school attendance/
30. 1 or 2 or 3 or 4 or 5 or 6 or 7 or 8 or 9 or 10 or 11 or 12
31. 13 or 14 or 15 or 16 or 17 or 18 or 19 or 20
32. 21 or 22 or 23 or 24 or 25 or 26 or 27 or 28 or 29
33. 30 and 31 and 32

For peer review only

Medline

1. child*.tw.
2. exp Child/
3. adolescen*.tw.
4. exp Adolescent/
5. student*.tw.
6. exp Students/
7. youth*.tw.
8. pupil*.tw.
9. schoolchild*.tw.
10. (young adj (people or person)).tw.
11. teen*.ti,ab.
12. p?diatric*.ti,ab.
13. exp suicide/
14. exp Suicide, Attempted/
15. exp Suicidal Ideation/
16. exp Drug Overdose/
17. exp Self-Injurious Behavior/ or exp Self Mutilation/
18. (autoaggress$ or auto aggress$ or automutilat$ or auto mutilat$ or cutt$ or overdose$ or (self adj2 cut$) or selfdestruct$ or self destruct$ or selfharm$ or self harm$ or selfimmolat$ or self immolat$ or selfinflict$ or self inflict$ or selfinjur$ or self injur$ or selfmutilat$ or self mutilat$ or selfpoison$ or self poison$ or suicid$).ti,ab.
19. (expulsion adj5 school*).ti,ab.
20. (Suspen* adj5 school*).ti,ab.
21. (manage* adj move*).ti,ab.
22. Terminat* adj5 school*).ti,ab.
23. (expel* adj5 school*).ti,ab.
24. ((exclusion or exclude*) adj6 school*).ti,ab.
25. ("failure to complete" adj5 (school* or educat*)).ti,ab.
26. ((school* or kindergarten or nursery or education*) adj4 (attend* or non-attend* or refus* or absen* or school phobi* or truan*)).tw.
27. 1 or 2 or 3 or 4 or 5 or 6 or 7 or 8 or 9 or 10 or 11 or 12
28. 13 or 14 or 15 or 16 or 17 or 18
29. 19 or 20 or 21 or 22 or 23 or 24 or 25 or 26
30. 27 and 28 and 29

For peer review only

ERIC

S47	S18 AND S43 AND S46
S46	S19 OR S20 OR S21 OR S22 OR S44 OR S45
S45	AB (autoaggress* OR "auto aggress*" OR automutilat* OR "auto mutilate*" OR cutt* OR overdos* OR (self N2 cut*) OR self-destruct* OR "self destruct*" OR selfharm* OR "self harm*" OR selfimmolat* OR "self immolate*" OR selfinflict* OR "self inflict*" OR selfinjur* OR "self injur*" OR selfmutilat* OR "self mutilate*" OR selfpoison* OR "self poison*" OR suicid*)
S44	AB (autoaggress* OR "auto aggress*" OR automutilat* OR "auto mutilate*" OR cutt* OR overdos* OR (self N2 cut*) OR self-destruct* OR "self destruct*" OR selfharm* OR "self harm*" OR selfimmolat* OR "self immolate*" OR selfinflict* OR "self inflict*" OR selfinjur* OR "self injur*" OR selfmutilat* OR "self mutilate*" OR selfpoison* OR "self poison*" OR suicid*)
S43	S23 OR S24 OR S25 OR S26 OR S27 OR S28 OR S29 OR S30 OR S31 OR S32 OR S33 OR S34 OR S35 OR S36 OR S37 OR S38 OR S39 OR S40 OR S41 OR S42
S42	DE "Withdrawal (Education)"
S41	DE "Attendance" OR DE "Average Daily Attendance" OR DE "College Attendance"
S40	DE "Suspension"
S39	DE "Expulsion"
S38	AB expel*
S37	TI expel*
S36	AB suspension
S35	TI suspension
S34	AB expulsion
S33	TI expulsion
S32	AB "failure to complete"
S31	TI "failure to complete"
S30	AB (exclusion OR exclude*)
S29	TI (exclusion OR exclude*)
S28	AB (terminat* N5 school*)
S27	TI (terminat* N5 school*)
S26	AB (manage* N1 move*)
S25	TI (manage* N1 move*)
S24	AB ((school* OR kindergarten OR nursery OR education*) N4 (attend* OR non-attend* OR refus* OR absen* OR school phobi* OR truan* OR dropout*))

For peer review only

S23	TI ((school* OR kindergarten OR nursery OR education*) N4 (attend* OR non-attend* OR refus* OR absen* OR school phobi* OR truan* OR dropout*))
S22	DE "Self Destructive Behavior"
S21	AB suicid*
S20	TI suicid*
S19	DE "Suicide"
S18	S1 OR S2 OR S3 OR S4 OR S5 OR S6 OR S7 OR S8 OR S9 OR S10 OR S11 OR S12 OR S13 OR S14 OR S15 OR S16 OR S17
S17	AB (young N1 (people OR person))
S16	TI (young N1 (people OR person))
S15	AB schoolchild*
S14	TI schoolchild*
S13	AB pupil*
S12	TI pupil*
S11	AB youth*
S10	TI youth*
S9	DE "Students"
S8	AB student*
S7	TI student*
S6	DE "Adolescents"
S5	AB adolescen*
S4	TI adolescen*
S3	AB child*
S2	TI child*
S1	DE "Child Health" OR DE "Children"

For peer review only

BEI

S49	S20 AND S25 AND S48
S48	S26 OR S27 OR S28 OR S29 OR S30 OR S31 OR S32 OR S33 OR S34 OR S35 OR S36 OR S37 OR S38 OR S39 OR S40 OR S41 OR S42 OR S43 OR S44 OR S45 OR S46 OR S47
S47	DE "SCHOOL dropouts"
S46	DE "COLLEGE attendance"
S45	DE "SCHOOL attendance"
S44	DE "STUDENT suspension"
S43	DE "EXCLUSION from school"

S42	DE "STUDENT expulsion"
S41	AB expel*
S40	TI expel*
S39	AB suspension
S38	TI suspension
S37	AB expulsion
S36	TI expulsion
S35	AB "failure to complete"
S34	TI "failure to complete"
S33	AB (exclusion OR exclude*)
S32	TI (exclusion OR exclude*)
S31	AB (terminat* N5 school*)
S30	TI (terminat* N5 school*)
S29	AB (manage* N1 move*)
S28	TI (manage* N1 move*)
S27	AB ((school* OR kindergarten OR nursery OR education*) N4 (attend* OR non-attend* OR refus* OR absen* OR school phobi* OR truan* OR dropout*))
S26	TI ((school* OR kindergarten OR nursery OR education*) N4 (attend* OR non-attend* OR refus* OR absen* OR school phobi* OR truan* OR dropout*))
S25	S21 OR S22 OR S23 OR S24
S24	AB (autoaggress* OR “auto aggress*” OR automutilat* OR “auto mutilate*” OR cutt* OR overdos* OR (self N2 cut*) OR self-destruct* OR “self destruct*” OR selfharm* OR “self harm*” OR selfimmolat* OR “self immolate*” OR selfinflict* OR “self inflict*” OR selfinjur* OR “self injur*” OR selfmutilat* OR “self mutilate*” OR selfpoison* OR “self poison*” OR suicid*)
S23	AB (autoaggress* OR “auto aggress*” OR automutilat* OR “auto mutilate*” OR cutt* OR overdos* OR (self N2 cut*) OR self-destruct* OR “self destruct*” OR selfharm* OR “self harm*” OR selfimmolat* OR “self immolate*” OR selfinflict* OR “self inflict*” OR selfinjur* OR “self injur*” OR selfmutilat* OR “self mutilate*” OR selfpoison* OR “self poison*” OR suicid*)
S22	AB suicid*
S21	TI suicid*
S20	S1 OR S2 OR S3 OR S4 OR S5 OR S6 OR S7 OR S8 OR S9 OR S10 OR S11 OR S12 OR S13 OR S14 OR S15 OR S16 OR S17 OR S18 OR S19
S19	AB (young N1 (people OR person))
S18	TI (young N1 (people OR person))
S17	AB schoolchild*

For peer review only

S16	TI schoolchild*
S15	AB pupil*
S14	TI pupil*
S13	AB youth*
S12	TI youth*
S11	DE "STUDENTS"
S10	AB student*
S9	TI student*
S8	DE "ADOLESCENT psychology"
S7	(DE "TEENAGERS") OR (DE "ADOLESCENCE")
S6	AB adolescen*
S5	TI adolescen*
S4	AB child*
S3	TI child*
S2	(DE "CHILD psychology") OR (DE "CHILD psychiatry")
S1	(DE "CHILDREN") OR (DE "CHILDREN -- Health")

For peer review only

Data Extraction

Reference:

Self-harm variable
Type and duration:

Binary/other:

Method of ascertainment:

Attendance/exclusion variable
Type and duration:

Binary/other:

Method of ascertainment:

Covariates:

N subjects (or cases/controls):

Characteristics of cohort:

Country:

Age (mean/median and range):

Study design:

Time period of data collection:

Odds ratio(s) or other effect estimate(s):

Confidence interval(s):

P Value(s):

Notes about results:

For peer review only

For quality assessment (See risk of bias tools for more information)

Cross sectional:

Representativeness of sample (0-1):
Sample size (0-1):
Non-respondents (0-1):
Ascertainment of attendance/exclusion variable (0-1):
Comparability (0-2):
Ascertainment of self-harm variable (0-1):
Statistical reporting (0-1):
Clarity of variable definitions (0-2):

Case control:

Case definition (0-1):
Representativeness of cases (0-1):
Selection of controls (0-1):
Definition of controls (0-1):
Comparability (0-2):
Ascertainment of attendance/exclusion variable (0-1):
Same ascertainment method for cases and controls (0-1):
Non-response rate (0-1):
Sample size (0-1):
Statistical reporting (0-1):
Clarity of variable definitions (0-2):

Cohort:

Representativeness of exposed cohort (0-1):
Selection of non-exposed cohort (0-1):
Definition of controls (0-1):
Ascertainment of attendance/exclusion variable (0-1):
Outcome not present at start (0-1):
Comparability (0-2):
Ascertainment of self-harm variable (0-1):
Follow-up long enough (0-1):
Adequacy of follow-up (0-1):
Sample size (0-1):
Statistical reporting (0-1):
Clarity of variable definitions (0-2):

For peer review only

Adapted Newcastle-Ottawa Quality Assessment Scales

Cohort Studies

Note: A study can be awarded a maximum of one point for each numbered item with the Selection and Exposure categories. A maximum of two points can be given for Comparability.

Selection (maximum 4)

1. Representativeness of the cohort
    a. Truly representative of the average in the target population (random sample or whole population)*
    b. Somewhat representative of the average in the target population (purposive sampling of representative schools or evidence that the sample is representative of the source population)*
    c. Selected group of users
    d. No description of the derivation of the cohort
2. Selection of the non-exposed cohort
    a. Drawn from the same community as the exposed cohort *
    b. Drawn from a different source
    c. No description of the derivation of the non-exposed cohort
3. Ascertainment of exposure (absence or exclusion)
    a. School administrative records*
    b. Reported by school staff*
    c. Other collateral or self-report
    d. No description
4. Demonstration that outcome of interest was accounted for or not present at start of study
    a. Yes *
    b. No

Comparability (maximum 2)

1. Comparability of cohorts on the basis of the design or analysis
    a. Study controls for age and gender (or analysis separated by gender) *
    b. Study controls for any additional factor *

Outcome (maximum 3)

1. Ascertainment of outcome (self-harm or suicidality)
    a. Structured interview or written self-report*
    b. Clinical record*
    c. Reported by school or collateral
    d. No description
2. Was follow-up long enough for outcomes to occur?
    a. Yes (>1 year) *
    b. No
3. Adequacy of follow-up of cohorts
    a. Complete follow-up – all subjects accounted for *
    b. Subjects lost to follow-up unlikely to introduce bias – small number lost (<20%) or attrition described and accounted for in analysis *
    c. Follow up rate not adequate and no description of those lost

For peer review only

d. No statement

Note: A study can be awarded a maximum of one point for each numbered item for sample size and statistical test. A maximum of two points can be given for clear variables.

Additional parameters: Not in NOS, taken from cross sectional NOS:

1. Sample size (Maximum 1)
    a. Justified and satisfactory *
    b. Adequately powered to detect a difference (at least 10 events per variable in multivariate analyses)*
    c. Not justified

2. Statistical test (Maximum 1)
    a. The statistical test used to analyse the data is clearly described and appropriate, and the measurement of the association is presented as either an OR, CI and P value or a beta coefficient, SE and P value*
    b. The statistical test is not appropriate, not described or incomplete

Further additional parameter:

1. Clear variables (Maximum 2)
    a. Both variables clearly defined including time period for measurement (2)
    b. One or other variable clearly defined including time period for measurement (1)
    c. Neither variable clearly defined including time period for measurement (0)

Case-Control Studies

Note: A study can be awarded a maximum of one point for each numbered item with the Selection and Exposure categories. A maximum of two points can be given for Comparability.

Selection (maximum 4)

5. Is the case definition adequate? (self-harm or suicidality)
    a. Structured interview or written self-report*
    b. Clinical record*
    c. Reported by school or collateral
    d. No description
6. Representativeness of the cases
    a. Consecutive or obviously representative series of cases *
    b. Potential for selection biases or not stated
7. Selection of controls
    a. Community controls from source population*
    b. Clinical controls if clinical source population*
    c. Not extracted from same source population
    d. No description
8. Definition of controls

For peer review only

a. No history of outcome (NB if cases have new (not necessarily first) occurrence of outcome, controls with previous occurrences should not be excluded) *
b. No description of source

Comparability (maximum 2)

2. Comparability of cases and controls on the basis of the design or analysis
    a. Study controls for age and gender (or analysis separated by gender)*
    b. Study controls for any additional factor *

Exposure (maximum 3)

4. Ascertainment of exposure (absence or exclusion)
    a. School administrative records*
    b. Reported by school staff*
    c. Other collateral or self-report
    d. No description
5. Same method of ascertainment for cases and controls
    a. Yes *
    b. No
6. Non-response rate
    a. Same rate for both groups *
    b. Non-response or missing values should be <20% and accounted for in analysis*
    c. Non respondents described
    d. Rate different and no designation

Note: A study can be awarded a maximum of one point for each numbered item for sample size and statistical test. A maximum of two points can be given for clear variables.

Additional parameters: Not in NOS, taken from cross sectional NOS:

3. Sample size (Maximum 1)
    a. Justified and satisfactory *
    b. Adequately powered to detect a difference (at least 10 events per variable in multivariate analyses)*
    c. Not justified

4. Statistical test (Maximum 1)
    a. The statistical test used to analyse the data is clearly described and appropriate, and the measurement of the association is presented as either an OR, CI and P value or a beta coefficient, SE and P value*
    b. The statistical test is not appropriate, not described or incomplete

Further additional parameter:

2. Clear variables (Maximum 2)
    a. Both variables clearly defined including time period for measurement (2)
    b. One or other variable clearly defined including time period for measurement (1)
    c. Neither variable clearly defined including time period for measurement (0)

For peer review only

Cross-Sectional Studies

Note: A study can be awarded a maximum of one point for each numbered item with the Selection and Exposure categories. A maximum of two points can be given for Comparability.

Selection (maximum 4)

9. Representativeness of the sample
    a. Truly representative of the average in the target population (random sample or whole population) *
    b. Somewhat representative of the average in the target population (purposive sampling of representative schools or evidence that the sample is representative of the source population) *
    c. Selected group of users
    d. No description of the sampling strategy
10. Sample size
    a. Justified and satisfactory *
    b. Adequately powered to detect a difference (at least 10 events per variable in multivariate analyses)*
    c. Not justified
11. Non-respondents
    a. Comparability between respondents and non-respondents characteristics is established, and the response rate is satisfactory (>60%)*
    b. The response rate is unsatisfactory, or the comparability between respondents and non-respondents is unsatisfactory
    c. No description of the response rate or the characteristics of the responders and non-responders
12. Ascertainment of the exposure (absence or exclusion)
    a. School administrative records*
    b. Reported by school staff*
    c. Other collateral or self-report
    d. No description

Comparability (maximum 2)

3. The subjects in different outcome groups are comparable, based on the study design or analysis. Confounding factors are controlled
    a. Study controls for age and gender (or analysis separated by gender)*
    b. Study controls for any additional factor *

Outcome (maximum 2)

7. Assessment of the outcome (self-harm or suicidality)
    a. Structured interview or written self-report*
    b. Clinical record*
    c. Reported by school or collateral
    d. No description

8. Statistical test

For peer review only

a. The statistical test used to analyse the data is clearly described and appropriate, and the measurement of the association is presented as either an OR, CI and P value or a beta coefficient, SE and P value*
b. The statistical test is not appropriate, not described or incomplete

---

Additional parameter not in NOS:

3. Clear variables (Maximum 2)
   a. Both variables clearly defined including time period for measurement (2)
   b. One or other variable clearly defined including time period for measurement (1)
   c. Neither variable clearly defined including time period for measurement (0)

For peer review only
